# Hydrogen-Terminated Two-Dimensional Germanane/Silicane Alloys as Self-Powered Photodetectors and Sensors

**DOI:** 10.1021/acsami.3c01971

**Published:** 2023-05-16

**Authors:** Pradip Kumar Roy, Tomáš Hartman, Jiří Šturala, Jan Luxa, Manuel Melle-Franco, Zdenek Sofer

**Affiliations:** † Department of Inorganic Chemistry, University of Chemistry and Technology Prague, Technická 5, 166 28 Prague 6, Czech Republic; ‡ CICECO—Aveiro Institute of Materials, Department of Chemistry, University of Aveiro, 3810-193 Aveiro, Portugal

**Keywords:** germanane and silicane, photoelectrochemical (PEC) photodetector, self-powered, vapor sensor, band bending

## Abstract

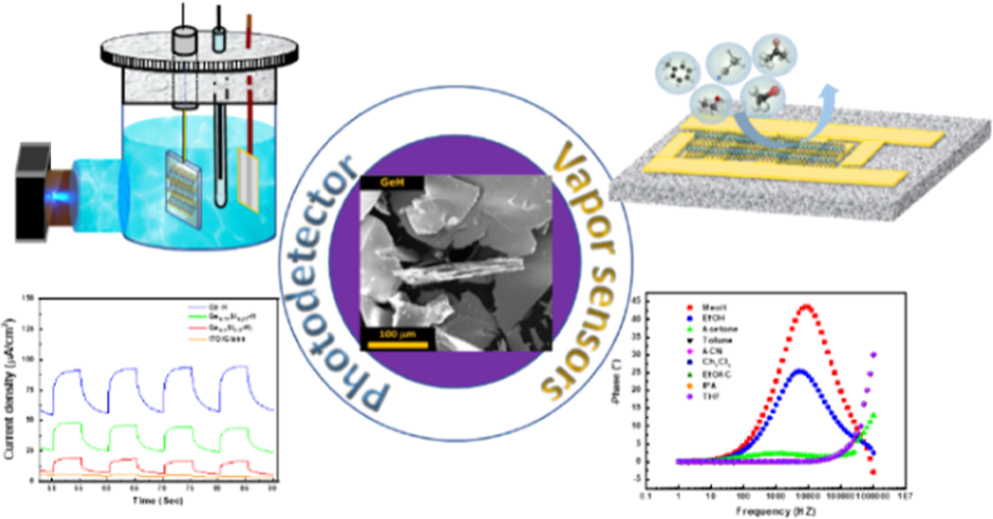

2D monoelemental materials, particularly germanene and silicene (the single layer of germanium and silicon), which are the base materials for modern electronic devices demonstrated tremendous attraction for their 2D layer structure along with the tuneable electronics and optical band gap. The major shortcoming of synthesized thermodynamically very unstable layered germanene and silicene with their inclination toward oxidation was overcome by topochemical deintercalation of a Zintl phase (CaGe_2_, CaGe_1.5_Si_0.5_, and CaGeSi) in a protic environment. The exfoliated Ge–H, Ge_0.75_Si_0.25_H, and Ge_0.5_Si_0.5_H were successfully synthesized and employed as the active layer for photoelectrochemical photodetectors, which showed broad response (420–940 nm), unprecedented responsivity, and detectivity on the order of 168 μA W^–1^ and 3.45 × 10^8^ cm Hz^1/2^ W^–1^, respectively. The sensing capability of exfoliated germanane and silicane composites was explored using electrochemical impedance spectroscopy with ultrafast response and recovery time of less than 1 s. These positive findings serve as the application of exfoliated germanene and silicene composites and can pave a new path to practical applications in efficient future devices.

## 1 Introduction

The last two decades have belonged to various 2D materials such as graphene, black phosphorus (BP), and transition-metal compounds due to their exceptional properties such as 2D quantum confinement and tuneable layer structure.^1−5^ These 2D materials offer a wide range of applications ranging from sensors, photodetectors, energy storage, catalysis to health monitoring.^1−3,6−13^ Graphene has been extensively studied due to its superior charge carrier mobility, thermal conductivity, and flexibility.^14^ However, its application is hampered by the lack of a band gap.^5^ Transition-metal dichalcogenides and BP are also attracting the attention of researchers, thanks to their tuneable band gap (0.3–2.0 eV) and moderate carrier mobility.^15−18^ The wide application of 2D transition metals and BP is severely limited by their low charge carrier mobility and poor environmental stability.^19−21^

2D monoelemental materials (Si, Ge, Sn, and other elements of the group 14) have emerged as the recent favorite of scientific research, thanks to their graphene-like structure, band opening due to spin–orbit coupling, tuneable band gap, and 2D quantum confinement effect, and are full of future potential.^22^ In particular, germanane and silicene (the single layer of germanium and silicon),^23,24^ which has mixed sp^2^/sp^3^ hybridization. The hydrogen termiated analogue GeH with a predicted compatible band gap of about 1.65 eV (GeH) have huge potential for optoelectronic applications exhibits superior carrier mobility and quantum Hall effect.^25−27^ In addition, silicon and germanium are the base materials for modern electronic devices and have a tremendous attraction for tuneable electronics and optical band gap due to their 2D layered structure of Si and germanene.^28,29^

This unprecedented opportunity for the future has led researchers to explore new germanane- and silicane-based materials. The synthetic protocol for germanane (GeH) or silicane (SiH) synthesis is facilitated by topochemical deintercalation of layered Zintl phase (CaSi_2_ or CaGe_2_, respectively) in aqueous HCl at low temperatures without the formation of germanene or silicene.^27^ The resulting structure of Si and Ge atoms shows an sp^3^-hybridized honeycomb-like structure analogous to graphene where each germanium is terminated mainly with hydrogen, whereas silicon also tends to form Si–OH/Si–O–Si bonds giving rise to geometry terminated with H/OH above or below the layer.^27,30^ However, compared to graphene, GeH or SiH is not planar. The pure silicane (SiH) lattice has a strong tendency to be oxidized and eventually forms polysiloxane under ambient conditions. For this reason, special care is required during the synthesis process represented by the oxygen-free environment.

So far, the focus on germananes/silicanes has been mainly on their stability and basic properties and only a few deals with their applications. Photocatalytic activity in hydrogen evolution was demonstrated using the covalently terminated germananes GeH and GeCH_3_.^31,32^ Song et al. employed functionalized germanene-based nanomaterials to detect Alzheimer’s disease-related single nucleotide polymorphisms using electrochemical impedance spectroscopy (EIS).^33^ For energy storage, a GeH-carbon composite was successfully used as an anode for lithium-ion batteries with a high energy density of ∼1100 mA h/g.^34^ The possibility of creating microrobots with different fluorescence emissions under UV light irradiation was investigated using functional 2D germanene.^35^ Finally, Si and Ge are known to be fully miscible to form Si_(1–*x*)_Ge_*x*_ alloy compounds with 0 < *x* < 1,^36^ that exhibit tuneable electronic and optical properties over a wide energy range. There are very few studies on the optoelectronic properties of Si_(1–*x*)_Ge_*x*_ alloy compounds, which provide us with a unique opportunity to explore the optical and electronic properties of Si_(1–*x*)_Ge_*x*_ alloy compounds.

Herein, we demonstrate the successful exfoliation of layered CaGe_2_, CaGe_1.5_Si_0.5_, and CaGeSi Zintl phases via low-temperature topochemical deintercalation providing GeH, Ge_0.75_Si_0.25_H, and Ge_0.5_Si_0.5_H, respectively (Figure S1). The exfoliated materials were thoroughly characterized by Raman spectroscopy, X-ray diffraction (XRD), X-ray photoelectron spectroscopy (XPS), Fourier transform infrared spectroscopy (FTIR), and thermogravimetric analysis (TGA). For the first time, exfoliated Ge–H, Ge_0.75_Si_0.25_H, and Ge_0.5_Si_0.5_H were successfully used for the photoelectrochemical (PEC)-based self-powered photodetector and organic vapor sensing.

## 2 Experimental Section

### 2.1 Materials

Silicon (99.999%), germanium (99.999%), and calcium (99.9%) were obtained from Alfa Aesar, Germany. Hydrochloric acid (37%) was obtained from Penta, Czech Republic.

### 2.2 Synthesis

The starting Zintl phase was prepared by the direct reaction of calcium, silicon, and germanium in the stoichiometric ratio in evacuated quartz ampoule with aluminum oxide liner. Calcium was used in 20 at. % excess to compensate losses by reaction with aluminum oxide and quartz glass at high temperature. Formed Zintl phases were mechanically removed from ampoule and stored in an argon-filled glovebox.

### 2.3 Instruments

The surface morphology of the exfoliated materials was investigated with scanning electron microscopy (SEM). An FEG electron source (Tescan Lyra dual beam microscope) was successfully used during the measurements with an applied voltage of about 15 kV electron beam.

The vibrational modes of GeH, Ge_0.75_Si_0.25_H, and Ge_0.5_Si_0.5_H were investigated using FTIR using an iS50R FTIR spectrometer (Thermo Scientific, USA) in the range of 2500–400 cm^–1^ studied.

XRD measurements were executed utilizing a Bruker D8 Discoverer (Bruker, Germany) powder diffractometer with a Cu Kα radiation source (λ = 0.15418 nm, *U* = 40 kV, and *I* = 40 mA). The measured XRD data were accumulated with an angular range of 5–90° (2θ) and a step size of 0.01517° (2θ). Finally, the XRD data were analyzed using High Score Plus software.

Themys TGA (SETARAM instrument) coupled with a mass spectrometer (OMNI star) was used for TGA of the exfoliated sample. The temperature range was between 30 and 600 °C with a heating rate of 10 °C min^–1^. During the measurement, helium was used as a carrier gas with a flow rate of 100 mL min^–1^.

Raman spectroscopy was performed using a Raman microscope (Renishaw) and a Charge Coupled Device (CCD) detector. A green DPSS laser (532 nm, 50 mW) was used as the laser source. The instrument was calibrated with the standard silicon peak (520 cm^–1^) and a resolution of less than 1 cm^–1^. We performed our experiments using the 20× objective of the microscope with a laser power of 0.5 mW and an exposure time of 25 s.

The XPS spectra were studied using a system SPECS, equipped with a monochromatic X-ray source XR 50 MF (1486.7 eV) and a Phoibos 150 hemispherical analyzer with 2D CCD detector. The measurement was performed in ultra-low vacuum (5 × 10^–10^ mbar or lower). High-resolution XPS of the exfoliated sample was performed using an ESCA Probe spectrometer (Omicron Nanotechnology Ltd, Germany) with a high-resolution scan of the desired nuclear lines at *E*_p_ = 20 eV. Wide-scan investigations were recorded at *E*_p_ = 40 eV. The sample was placed on a highly conductive sample stage (a high purity gold bar). During the measurement, the charging effect was removed with an electron gun (1–5 V).

An Autolab PGSTAT 204 (Metrohm, Switzerland) was used to perform all electrochemical characterizations and photosensor studies. PEC photodetection was performed using a three-electrode system [deposited materials: working electrode (WE); Pt: counter electrode; and saturated calomel electrode (SCE): reference electrode].

The vapor sensing measurement was performed using a prefabricated Au-based finger electrode system. Impedance spectroscopic (EIS) measurements were performed using an Autolab (PGSTAT204) FRA32M impedance module. The measured frequency ranged from 0.01 Hz to 1 MHz with a logarithmic scale of 10 points per decade.

The SZ-05-H3 LED module was used for illumination, which produces a light output of 144 lumens at a current of 500 mA and can reach up to 244 lumens when operated at the maximum current of 1000 mA. Throughout the experiment, a proper cooling system was used to keep the power constant. The amazing light output is achieved by connecting four closely spaced LUXEON Z-LEDs LXZ1-PB01 in series, soldered on a thermally efficient 20 mm wide and 1.6 mm thick MCPCB aluminum base.

### 2.4 Computer Models

DFT calculations were performed with the QuantumATK version U-2022.12 simulation package.^62^ with a periodic unit cell of approximately 8 × 8 × 30 nm^3^ containing 8 Ge/Si atoms and 8 H atoms with 4 × 4 points in the 2D reciprocal space and the HSE06 functional. PseudoDojo norm-conserving fully relativistic pseudopotentials^63^ and the medium numerical basis sets were used for all atoms. This methodology was chosen since it was found to reproduce the experimental lattice dimensions of Ge and Si crystals with an accuracy slightly better than 0.5%.

## 3 Results and Discussion

The synthesis of layered Ge/Si composites was initially justified by the preparation of the corresponding Zintl phase, in this case CaGe_2_, CaGe_1.5_Si_0.5_, and CaGeSi. These Zintl phases were previously synthesized but not exfoliated by Vogg et al.^30^ and characterized. It is important to note that the quality of the Zintl phase is extremely important for the high quality of the exfoliated materials. The quality of the Zintl phase was characterized by Raman spectroscopy, XRD analysis, and XPS, as shown in Figures S2–S6. The results clearly demonstrate the excellent quality of the initial Zintl phases and are in the agreement with the previously published results.^27,30,37^

**Figure 1:**
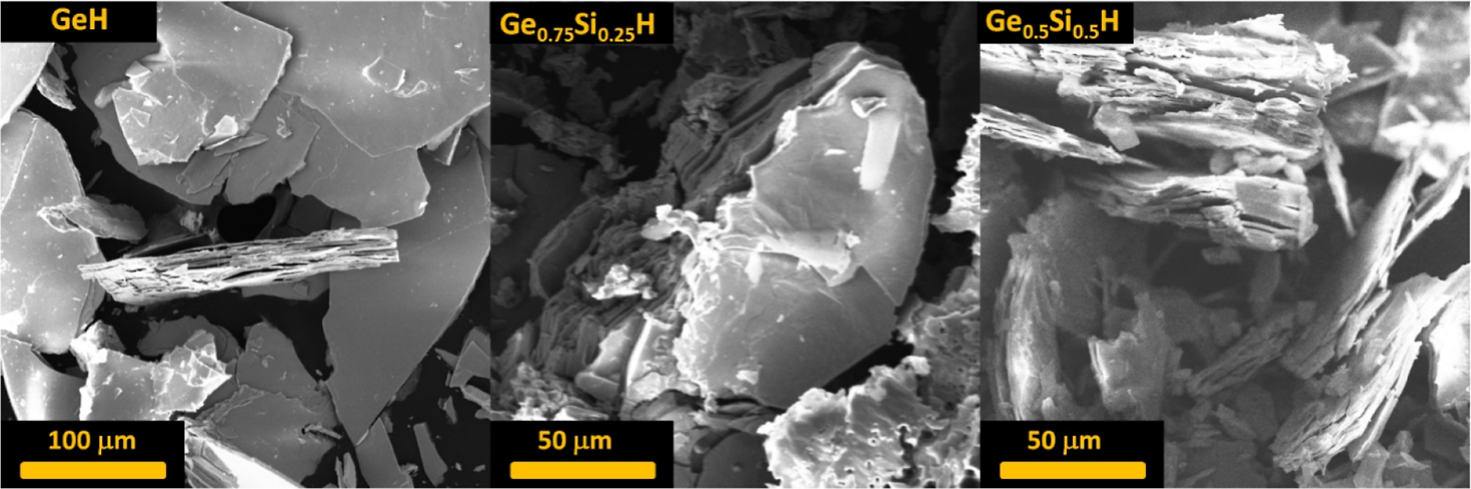
Top-view SEM image of Ge–H, Ge_0.75_Si_0.25_H, and Ge_0.5_Si_0.5_H.

**Figure 2:**
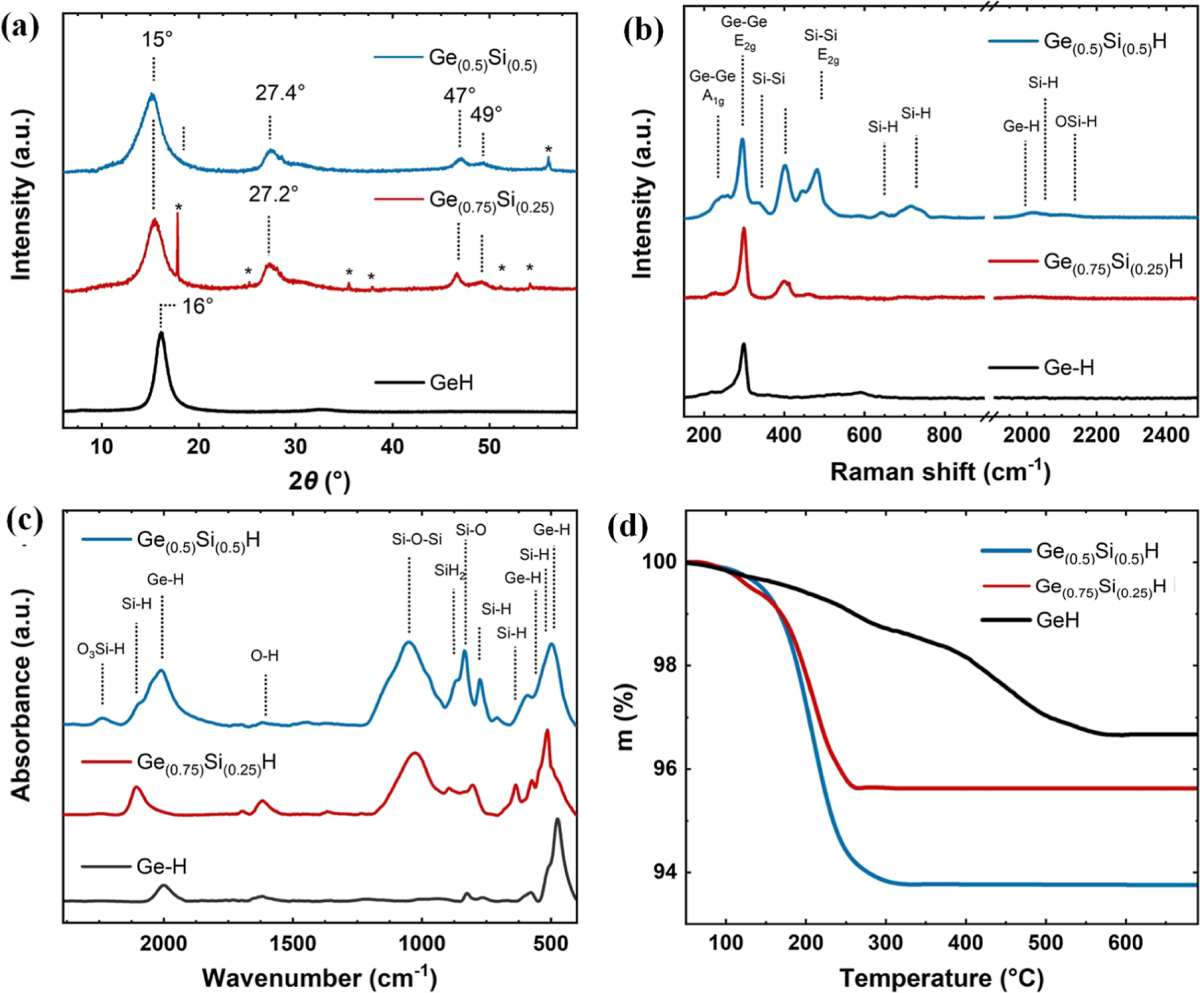
Crystal structure: (a) XRD patterns; asterisks label traces of silicon impurity, (b) Raman spectra, (c) FTIR, and (d) TGA of exfoliated Ge–H, Ge_0.75_Si_0.25_H, and Ge_0.5_Si_0.5_H.

**Figure 3:**
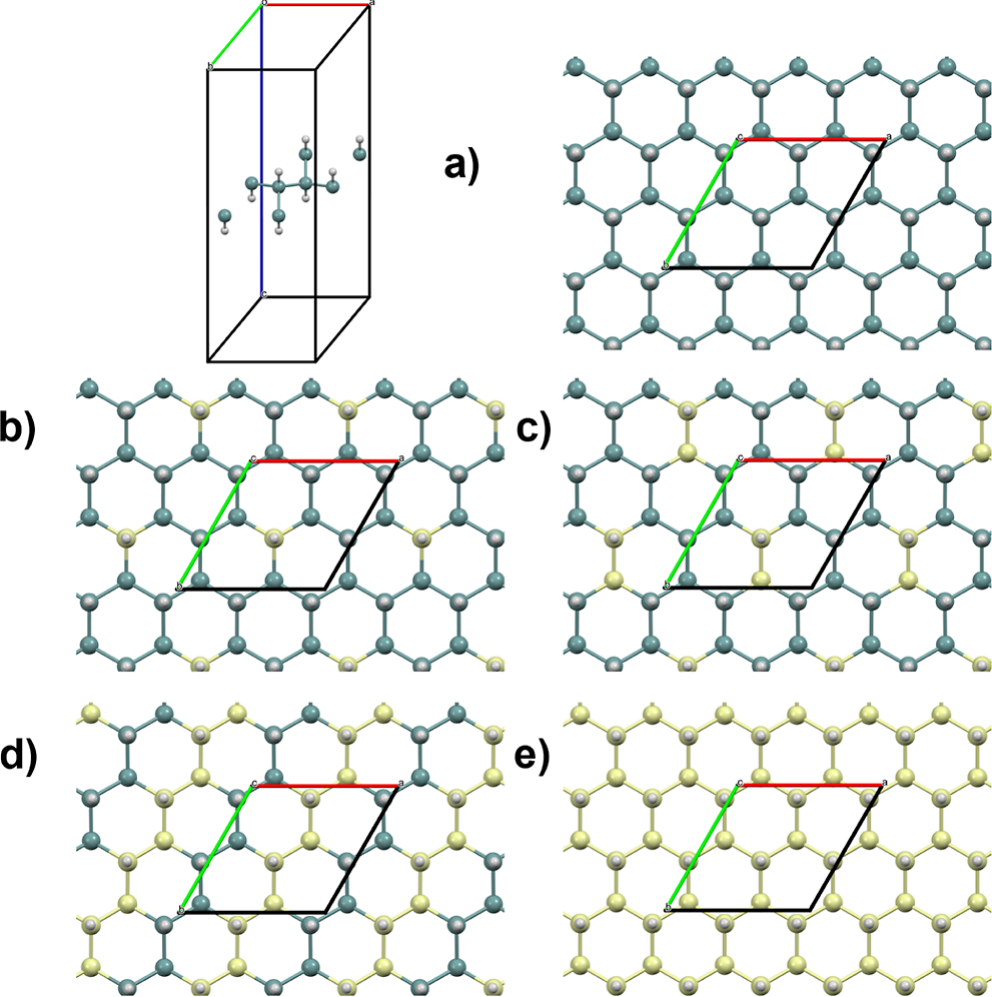
DFT, HSE06/medium-PseudoDojo level, periodic models of (a) GeH, two views, (b) Ge_0.825_Si_0.125_H, (c) Ge_0.75_Si_0.25_H, (d) Ge_0.5_Si_0.5_H, and (e) SiH.

The morphology of exfoliated Ge–H, Ge_0.75_Si_0.25_H, and Ge_0.5_Si_0.5_H was characterized by SEM, as shown in Figure 1, which clearly demonstrates the layered morphology of the materials. Electron-dispersive X-ray spectroscopy elemental mapping of the exfoliated materials shows a uniform distribution of elements throughout the material (Figure S7). The flake thickness of the exfoliated Ge–H, Ge_0.75_Si_0.25_H, and Ge_0.5_Si_0.5_H materials was determined by AFM, as shown in Figure S8a–i. After exfoliation of the material, we observed flakes with a relatively small lateral size (∼0.4 μm) but with thickness up to 15 nm. We constructed a thickness profile of the exfoliated flakes (see Figure S8), in which many nano flakes were observed. The XRD results are displayed in Figure 2a. The exfoliated materials show much broader peaks compared to the Zintl initial phase (Figure S2, Table S1–S3). This phenomenon indicates that the exfoliated materials are dominated by an amorphous phase in terms of interlayer spacing and layer number. The main peak of Ge–H at 2θ–16° corresponds to the 002 plane (∼5.5 Å) and with increasing silicon content, the 002 reflection shifts further to ∼15° (∼5.7 Å) for Ge_0.75_Si_0.25_H and ∼5.8 Å for Ge_0.5_Si_0.5_H. The peaks at 2θ–27, 47, and 49° correspond to the 100, 110, and 112 plane,^27^ and the 100 reflection at ∼26.5° (for GeH), ∼27.2° (for Ge_0.75_Si_0.25_H), and ∼27.4° (for Ge_0.5_Si_0.5_H), respectively, which represent the *a*-parameter of the lattice for Ge–H, Ge_0.75_Si_0.25_H, and Ge_0.5_Si_0.5_H, which are ∼3.90 Å for GeH, ∼3.78 Å for Ge_0.75_Si_0.25_H, and ∼3.76 Å for Ge_0.5_Si_0.5_H, respectively, indicating a small contraction of the unit cell with the increasing silicon content. Hybrid DFT computer models reveal that GeH 2D unit cell is slightly longer than the SiH 2D unit cell, with values of 3.98 and 3.88 Å, respectively. This matches well the trend of the 3D related pure materials, α-Ge and α-Si, which show comparable values for the rhombohedral primitive unit cell lattice dimensions, 3.99 and 3.86 Å, respectively. The lattice mismatch accounts for the progressive reduction of the 2D material dimensions with the increase of the Si content. This was also explicitly verified by computing Ge_0.825_Si_0.125_H, Ge_0.75_Si_0.25_H, and Ge_0.5_Si_0.5_H (Figure 3), which yielded unit cells with corresponding lengths of 3.96, 3.95, and 3.92 Å, respectively. Furthermore, these models also indicate that Si doping on GeH is mildly exothermic with respect to GeH and SiH, namely, 6, 7, and 8 meV per Ge/Si atom for Ge_0.875_Si_0.125_H, Ge_0.75_Si_0.25_H, and Ge_0.5_Si_0.5_H, respectively.

Raman spectroscopy was used to study starting Zintl phases as well as the exfoliated materials. The successful exfoliation of the Zintl phases was confirmed by the fact that the Raman peaks of the Zintl phases disappeared, and new peaks appeared in the spectrum (Figure 2b). For GeH, the prominent peak is at about 300 cm^–1^, which can be attributed to the *E*_2g_ vibrational mode (Ge–Ge in the plane) and is most dominant, as well as for Ge_0.75_Si_0.25_H and Ge_0.5_Si_0.5_H. The new peak at ∼ 400 cm^–1^ was found for the exfoliated Ge_0.75_Si_0.25_H Si–Si vibrational mode.^27^ A new *E*_2g_ (Si–Si) vibrational mode was observed for Ge_0.5_Si_0.5_H along with peaks at ∼650 and ∼700 cm^–1^ assigned to the Si–H vibrational modes.^27^ Three weak peaks were also observed for the vibrational mode at ∼ 2000–2200 cm^–1^ corresponding to Ge–H, Si–H, and O–Si–H, as shown in Figure 2b.

**Figure 4:**
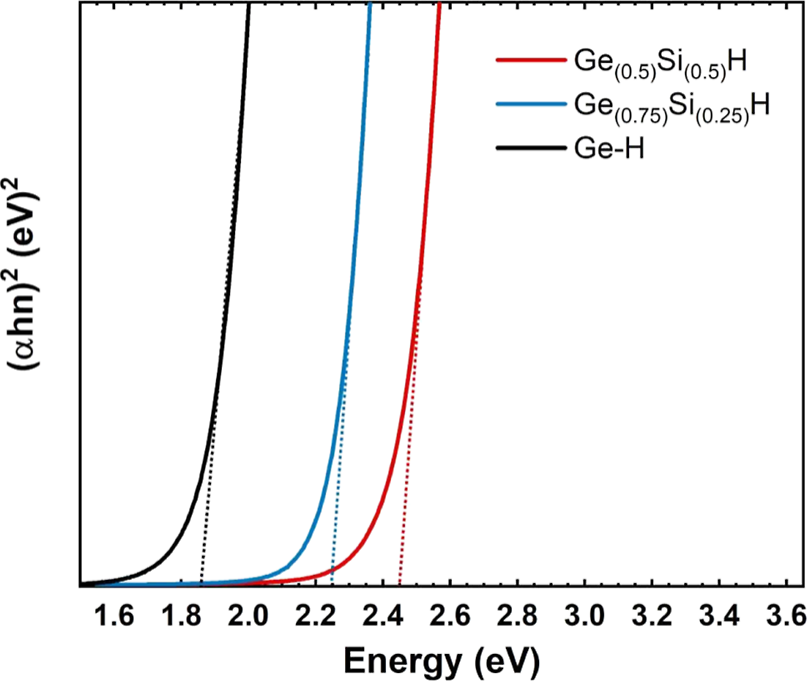
Tauc plot and corresponding band gap of exfoliated Ge–H, Ge_0.75_Si_0.25_H, and Ge_0.5_Si_0.5_H.

FTIR was used to study the characteristic vibrational modes of exfoliated germanane and its silicon composites, as shown in Figure 2c. Germanane exhibits two main peaks at ∼2000 and ∼500 cm^–1^, which can be assigned as stretching and wagging Ge–H vibrational modes. Interestingly, the peak at ∼500 cm^–1^ can be deconvoluted into three other peaks 574, 502, and 475 cm^–1^ associated with the Ge–H wagging vibrations.^27,37^ The other two vibrations registered below 900 cm^–1^ are associated with the bending Ge–H_2_ modes from the sheet’s edges or at defects.^38,39^ We have not observed any Ge–O–Ge or Ge–O vibrational modes for the Ge–H occurring between 800 and 1000 cm^–1^.^40^ Ge_0.75_Si_0.25_H and Ge_0.5_Si_0.5_H showed peaks at ∼2050 and 2150 cm^–1^ corresponding to Si–H, OSi–H stretching vibrations. Bending and wagging Si–H vibrations are also visible at ∼600–900 cm^–1^. Very intensive vibrational modes for the exfoliated Ge_0.75_Si_0.25_H and Ge_0.5_Si_0.5_H appear between 800 and 1000 cm^–1^ which correspond to various Si–O modes. The temperature stability of GeH, Ge_0.75_Si_0.25_H, and Ge_0.5_Si_0.5_H was investigated by TGA in an inert atmosphere (Figure 2d). GeH exhibits a mass loss of ∼1.1% at 200–250 °C, which is similar to the mass loss of 1 equiv of hydrogen, possibly from the more reactive sites, e.g., the edges or the more exfoliated fractions.^27,36^ The second mass loss was recorded at 400–500 °C, which probably corresponds to the loss of Cl (3.6 mol %), as described in the earlier report.^27^ Exfoliated Ge_0.75_Si_0.25_H and Ge_0.5_Si_0.5_H registered a noticeable mass loss of ∼4.3 and 6.3%, respectively, which can be attributed to the loss of H_2_O and hydrogen as it is present in larger amounts (Figure 2d). The optical properties of the materials was studied with the Tauc plot based on the absorbance measurement of UV–vis spectra (Figure 4). The smallest band gap was found for pure GeH ∼1.81 eV, followed by Ge_0.75_Si_0.25_H ∼ 2.25 eV and Ge_0.5_Si_0.5_H ∼ 2.45 eV. A similar trend was observed on the DFT computed band gaps which also increase with the doping degree, passing from 2.23 eV, for GeH, followed by Ge_0.75_Si_0.25_H, 2.53 eV and Ge_0.5_Si_0.5_H, 2.67 eV until the predicted saturation value of 2.90 eV for SiH (Figure S9).

**Figure 5:**
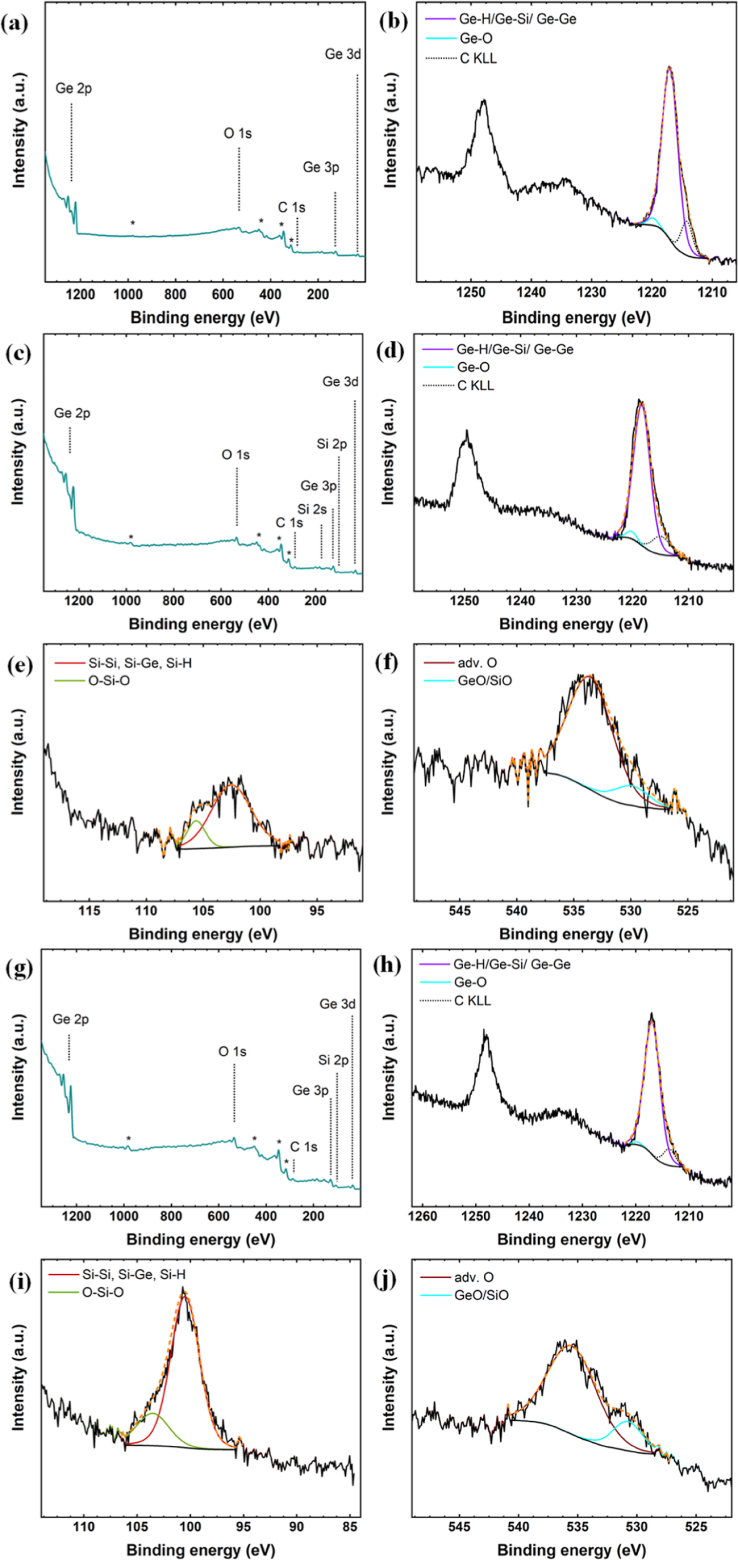
XPS spectra: (a,c,g) survey spectra of Ge–H, Ge_0.75_Si_0.25_H, and Ge_0.5_Si_0.5_H, respectively. (b,d–f,g–j) High-resolution spectra of the corresponding elements of exfoliated Ge–H, Ge_0.75_Si_0.25_H, and Ge_0.5_Si_0.5_H, respectively.

The elemental composition and chemical state of exfoliated GeH, Ge_0.75_Si_0.25_H, and Ge_0.5_Si_0.5_H were investigated by the XPS measurement. The wide survey and high-resolution analysis of the XPS spectrum are shown in Figure 5a,b, c–f and g–j for Ge–H Ge_0.5_Si_0.5_H and Ge_0.75_Si_0.25_H, respectively. The high-resolution spectrum of Ge 2p region can be decomposed into two peaks, i.e., the peak at 1217.8 eV corresponds to the Ge–H/Ge–Si/Ge–Ge and Ge–O at ∼ 1220 eV. The high-resolution spectrum of Si 2p consists of two deconvoluted peaks at ∼100 and ∼104 eV, corresponding to the Si–Si/Ge–Si/Si–H and Si–O bonding states, respectively. Finally, the high-resolution analysis of the O 1s region contains two fully resolved peaks corresponding to the Si–O/Ge–O and adventitious oxygen (Adv-O) bonding states at ∼535 and 531 eV, respectively.

The PEC properties of germanane and silicane composites have been carried out in different electrolyte environments such as acidic, basic, ionic, and neutral. It is important to point out that the materials demonstrated stable response at high power density, which differ from recent publication,^41^ and may be attributed to the superior crystal properties of the material. Photodetector performances were investigated using a three-electrode system, as shown in Figure S10. First, the I-V characteristics of the GeH, Ge_0.75_Si_0.25_H, and Ge_0.5_Si_0.5_H-based PEC photodetector were investigated using linear sweep voltammogram with a scanning speed of 10 mV s^–1^ under the illumination of 420 nm LED, as shown in Figure S11. The results show a continuous increase in current density with the increase of the applied voltage, indicating a clear response when the 420 nm LED light source is turned on and off. Moreover, the result shows that the current increases exponentially after 0.8 V versus SCE. As a precautionary measure, the applied potentials were limited to +0.5 V vs SCE to avoid oxidative conditions that could lead to uncontrolled (photo) electrochemical degradation of the photoelectrodes. The power dependence photoresponse of Ge–H in KOH solution is shown in Figure S12a at an applied voltage of 0.5 V against a SCE under the illumination of 420 nm LED. The current density increases continuously from 2 to 37 μA cm^–2^ with increasing power from 60 to 800 mW. This obvious phenomenon is due to the increasing generation of electron holes, which leads to an increase in current density. As can be seen in Figure S12b,c, the photoresponse properties can also be applied to different wavelengths of light. Similarly, the photoresponse using 420 nm LED increases with increasing power from 60 to 800 mW.

**Figure 6:**
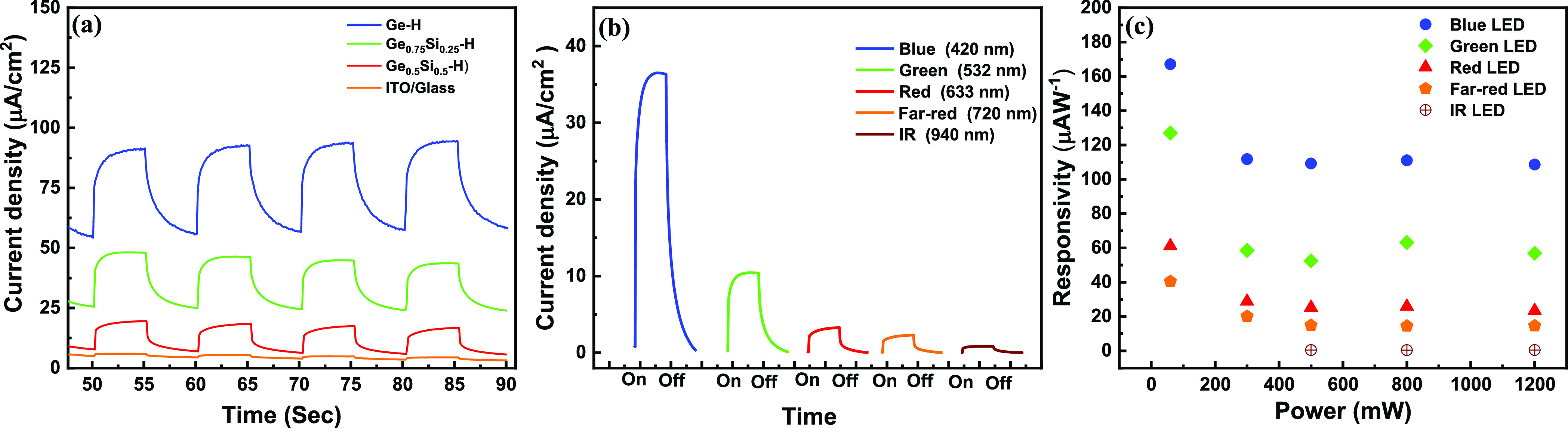
PEC photodetector properties of exfoliated materials. (a) Current density of GeH compared to Ge_0.75_Si_0.25_H and Ge_0.5_Si_0.5_H. (b) Current density of exfoliated GeH at different wavelengths. (c) Photoresponsivity of GeH at different powers measured in 1 M KOH.

We also studied the photoresponse of Ge_0.75_Si_0.25_H and Ge_0.5_Si_0.5_H in KOH solution at an applied voltage of 0.5 V against a SCE under the illumination of 420 nm LED and compared with GeH, as shown in Figure 6a. The trend clearly confirms that the current density is best for GeH and decreases from Ge_0.75_Si_0.25_H to Ge_0.5_Si_0.5_H. The reason is that with the incorporation of Si, the composite tends to interact with O_2_ and forms a Si–O–Si bond, which further increases the possibility of degradation of the composite sample, so the conductivity of the sample tends to decrease with increasing Si concentrations. The photoresponse of the GeH-based photodetector was also studied with different illumination wavelengths from blue (420 nm) to IR (940 nm) (Figure 6b). The results showed the highest response applying 420 nm light, followed by green (532 nm) and red (633 nm) illumination. Although the response of the photodetector is much lower with 940 nm illumination, it has a pronounced response from blue to IR light, making it suitable for broadband photodetection applications.

The photodetection performance of the PEC photodetector has been carried out by assessing different essential parameters. One of the most valuable parameters is responsivity (*R*_ph_), which is the ratio between the output current with respect to the power of the input irradiation signal, and can be evaluated using the following equation: *R*_ph_ = Δ*I*/*P*. Here, Δ*I* represents the change in current of the device under dark and the light illumination, whereas *P* corresponds to the irradiation power intensity per unit area. The calculated results for GeH exhibited height responsivity of 168 μAW^–1^ under the illumination with 420 nm light (Figure 6c). The evaluated responsivity (*R*_ph_) of the GeH photodetector device with different illumination wavelength and power has been highlighted in Figure 6c. The assessed results prominently described highest *R*_ph,_ for blue LED and decreased gradually for green to IR illumination. The calculated results are highlighted in Table S4, which is better or comparable with the published results.^20,42−51^

Followed by the responsivity, we have evaluated another important parameter in the form of specific detectivity, which can be determined by the formula described below^52^
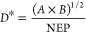
1where *A*, *B*, and NEP represents the active area of the photodetector device, measured bandwidth, and noise equivalent power, respectively. NEP is the signal power that produces a signal-to-noise ratio and equals to 1, representing the minimum impinging optical power that a photodetector can distinguish from the noise. Additionally, NEP can be represented as the formula described below^52,53^
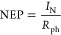
2where *R*_ph_ and *I*_N_ correspond to the photoresponsivity of the photodetector device and the noise current, respectively. Furthermore, the noise, which is in closely approximated with the dark current (*I*_D_) of the photodetector device, can be described as
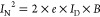
where “*e*” is the electronic charge.^54^ After combining eqs 1 and 2, the calculated specific detectivity of our photodetector devices evaluated is about 3.45 × 10^8^ cm Hz^1/2^ W^–1^. The change in specific detectivity with power and different illumination wavelengths have been depicted in Figure S13a.

**Figure 7:**
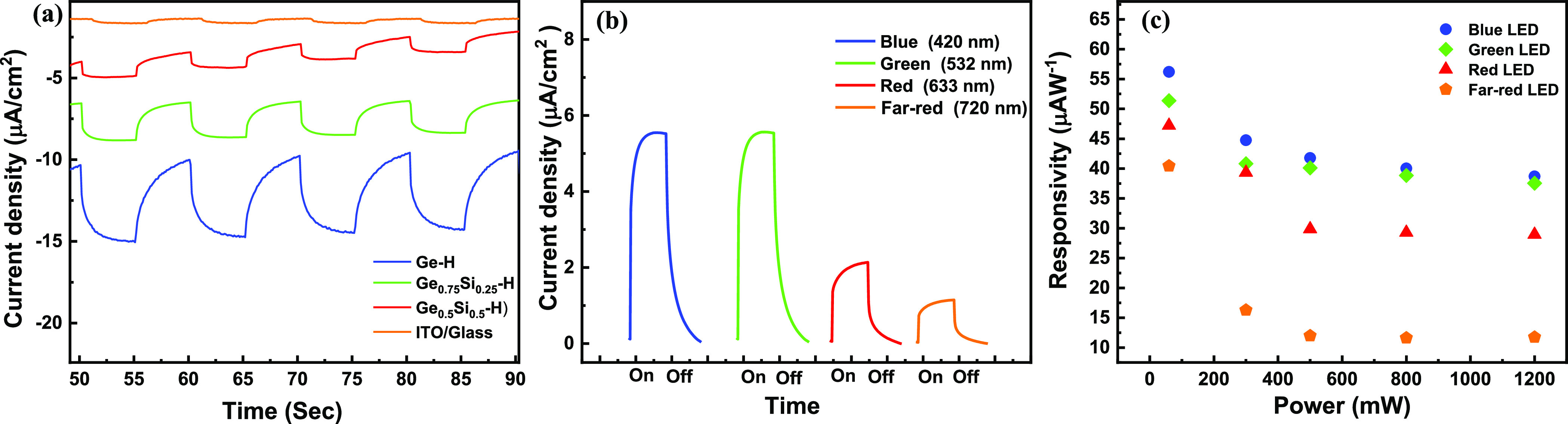
PEC photodetector properties of exfoliated materials. (a) Current density of GeH compared to Ge_0.75_Si_0.25_H and Ge_0.5_Si_0.5_H. (b) Current density of exfoliated GeH at different wavelengths. (c) Photoresponsivity of GeH at different powers measured in 0.5 M H_2_SO_4._

The photoresponse properties of GeH, Ge_0.75_Si_0.25_H, and Ge_0.5_Si_0.5_H were also investigated in 0.5 M H_2_SO_4_ solution at an applied voltage of 0.5 V vs SCE under the illumination of 420 nm LED with the highest response to GeH (Figure 7a). The photoresponse of the photodetectors was also performed with different illumination wavelengths. As with the KOH solution, there is a high response at 420 nm and an almost similar response with green illumination and then a continuous decrease at red and far red (720 nm) LED illumination, as shown in Figure 7b. Here, there is no response at IR illumination, although the response from green to far red is clear. The calculated photoresponsivity with different powers and wavelengths is shown in Figure 7c, which shows a height responsivity of 56 μA W^–1^ at an applied voltage of −0.5 Vs SCE under the illumination of 420 nm LED, which is lower compared to the KOH solution but still comparable to the published results (Supporting Information Table S4). The specific detectivity of the photodetector in 0.5 M H_2_SO_4_ solution was calculated and is plotted against the irradiance in Figure S13b, which shows a height value on the order of 1.16 × 10^8^ cm Hz^1/2^ W^–1^. We extended our study to neutral and ionic electrolytes, as shown in Figures S14 and S15, respectively. The results show a pronounced on/off response at different powers and wavelengths, proving the functionality of our photodetectors in acidic, alcoholic, ionic, and natural solutions. The photoresponse also follows the same trend, showing a better response to GeH compared to Ge_0.75_Si_0.25_H and Ge_0.5_Si_0.5_H.

Another important parameter of a photodetector is its response time. We calculated the response time of GeH in the Na_2_SO_4_ electrolyte under illumination with 420 nm light (500 mW) with a change in current density from 10 to 90%. The results shown in Figure S16 indicate ultrafast rise and fall times of 80 and 700 ms, respectively. We also investigated the response time of the GeH photodetector in ionic and KOH electrolytes (Figure S17). It is interesting to note that the response time is much faster in ionic and neutral solution compared to KOH solution, which might be due to the preferential and fast electron transfer in ionic or neutral solution.

The self-powered capability of the photodetector device was investigated by measuring the photoresponse at 0 V of applied voltage versus SCE (see Figure S18). However, in electrochemistry, the voltage at the WE is always measured in comparison to the reference electrode. Therefore, it is very difficult to confirm that the voltage at the WE is exactly zero. For this reason, we also measured the open-circuit potential of the photodetector, which corresponds to the build-up of the potential due to the banding of the materials in the depleted electrolyte. As can be seen in Figure S19a, the Fermi level of the material and the solution were not at the same energy level before immersion in the solution. Once we immersed the semiconductor in the solution, band bending occurs due to charge transfer to match the Fermi level of the semiconductor to that of the solution, resulting in charge separation within the semiconductor and further potential build-up in the semiconductor.^55−57^ Here, n-type Ge–H is immersed in KOH solution, which exhibits upward band bending, as shown in Figure S19b. The band bending causes positive charge to accumulate on the surface of the semiconductor, while *e*– migrates in the opposite direction due to the band bending and builds up a potential in the semiconductor. The band bending gradually returns to its original position when light is incident due to the reverse band bending (Figure S19c). This build-in potential can be measured experimentally via the open-circuit potential, as described in Figure S20, which was demonstrated to be a build-in potential on the order of 30 mV in 1 M KOH solution under the illumination of 420 nm LED (800 mW), confirming the self-powering capability.

The stability of the photodetector is another important parameter for its practical application. We investigated the time-dependent photocurrent response of the GeH, Ge_0.75_Si_0.25_H, and Ge_0.5_Si_0.5_H photodetector in 1 M KOH solution under the illumination of 420 nm LED (500 mW), as shown in Figures S21 and S22. The result shows a quite stable response that can be switched efficiently during the on and off switching process, with a negligible drop after a consecutive switching of more than 2000 s. A slight decrease in the photoresponse can be attributed to the sample loss during the measurement. The long-term stability of the photodetector was also tested, as shown in Figure S23, which shows a fairly stable response after 5 days.

**Figure 8:**
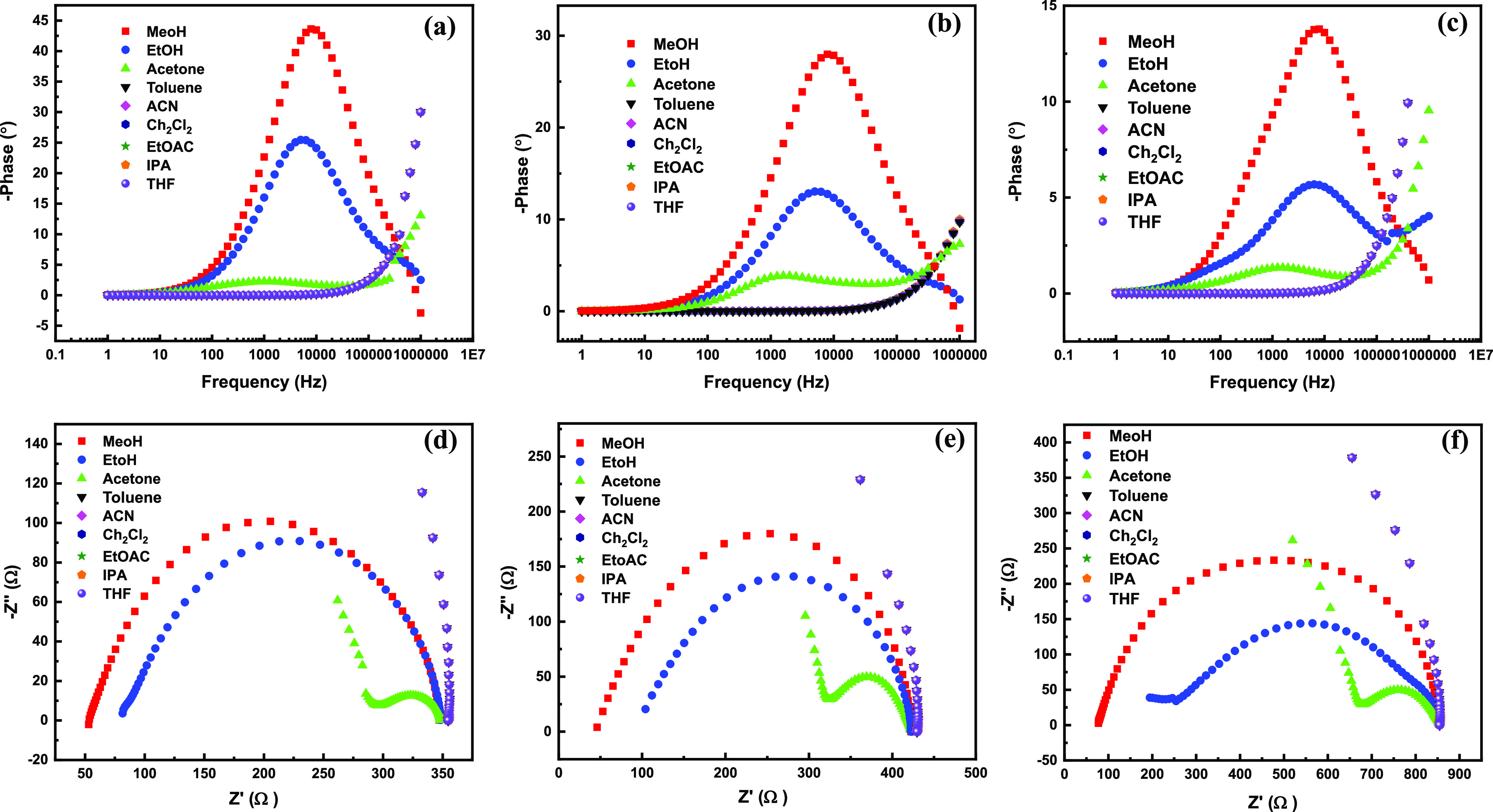
Vapor sensor performance. (a–c) Bode plot and (d–f) Nyquist plot for Ge–H, Ge_0.75_Si_0.25_H, and Ge_0.5_Si_0.5_H, respectively.

Selective and fast vapor sensors are of greatest interest for the deployment of advanced sensors using 2D materials, which provide a favorable platform due to their large surface-to-volume ratio and suitable band alignments. Here, we have successfully exploited the sensing capability of exfoliated germanane and silicane composites using EIS, which is a powerful method for surface characterization and sensing applications.^58,59^ The materials were sprayed onto a prefabricated gold electrode and then dried in an oven at 60° for 30 min to establish good contact between the exfoliated material and the electrode surface (Figure S24). The sensitivity of our devices was studied under the same exposure to concentrated organic vapor solutions. The volatile organic molecules were specifically selected because they are of particular interest in biochemistry and health care due to their occurrence in human exhalation and are in high demand in food and industrial production.^60,61^ The detection mechanism for organic molecules is based on the change of impedance response due to the change of local charge carrier concentration in 2D materials, which occurs due to the adsorption of molecules on the material surface (Figure S24). The intensity of the impedance signals of the organic molecules is determined by the strength of the interaction. The sensor response of the devices was investigated in a frequency range from 1 to 10^6^ Hz under organic saturated vapor arrangement. Ge–H and Ge_0.75_Si_0.25_H sensors show a pronounced response at a frequency around 8.5 MHz for methanol vapor, shifted slightly to 7.5 MHz for Ge_0.5_Si_0.5_H (Figure 8a–c). The peak position for ethanol and acetone vapor is almost unchanged at 5.2 and 1 MHz, respectively. A significant change in the phase was registered, decreasing continuously from Ge–H to Ge_0.5_Si_0.5_H specifically for methanol and ethanol vapor, while the change in response for acetone from Ge–H to Ge_0.5_Si_0.5_H was almost negligible. The performance of the sensor is also evidenced by the Nyquist plots (see Figure 8d–f), which confirm that the impedance of methanol, ethanol, and acetone vapors increased from 300, 275, 65 Ω to 377, 324, 100 Ω for Ge–H and Ge_0.75_Si_0.25_H, respectively, while it further increased to 780, 600, and 170 Ω for Ge_0.5_Si_0.5_H (methanol, ethanol, and acetone, respectively). The individual response of different saturated vapors to Ge–H, Ge_0.75_Si_0.25_H, and Ge_0.5_Si_0.5_H is depicted in Figure S25, which shows a pronounced response to methanol, ethanol, and acetone among all other organic vapors. The important parameter in the form of response time of the sensor was investigated, as shown in Figure S26, which shows excellent fast response with less than 1 s (Figure S26b). In addition, the sensor reaches its original current density within 1 s (Figure S26c), without heating or other external disturbances. The sensing performance of the device was illustrated by an equivalent circuit, as shown in Figure S27, where the sensing response can be simplified by three resistor–capacitor circuit (RC) circuits connected in parallel.^13^ The two RC circuits on the left and right sides (Figure S27) represent the contact point between the surfaces of the exfoliated material and the Au finger electrode, while the middle RC circuit represents the interaction between the layers of the 2D materials. Finally, the long-term stability of the sensor device was also verified, as shown in Figure S28, which shows a quite stable response after 5 days.

## 4 Conclusions

We have successfully exfoliated Ge–H, Ge_0.75_Si_0.25_H, and Ge_0.5_Si_0.5_H from the corresponding Zintl phases (i.e., CaGe_2_, CaGe_1.5_Si_0.5_, and CaGeSi). The hydrogen-terminated two-dimensional germanane and silicane composites were used as active materials in PEC-based self-powered photodetectors and were found to exhibiting broadband response with unprecedented sensitivity and detectivity on the order of 168 μA W^–1^ and 3.45 × 10^8^ cm Hz^1/2^ W^–1^, respectively, for Ge–H in KOH solution at an applied voltage of 0.5 V (Vs SCE) with illumination of 420 nm LED. The photodetector performance of the two-dimensional hydrogen-terminated germanane and silicane composites was also successfully carried out in other electrolyte environments such as acidic, ionic, and neutral solvents with stable photoresponse. The self-power capability of the photodetector was investigated in detail by semiconductor band bending in solution. The composite of exfoliated germanane and silicane has shown excellent vapor sensing capability with ultrafast response and recovery time of less than 1 s by EIS.

## Supporting Information Available

Characterization of Zintl phases (Raman, XRD, and XPS), SEM, EDX, AFM, schematic of the photodetector device, IV characteristic curve, detectivity of PEC-type photodetectors, photoresponse of photodetector in Na_2_SO_4_ and ionic solutions, comparison, schematic of semiconductor band bending, open-circuit potential, stability of the photodetector, response time, and RC equivalent circuit (PDF)
