# Supplementary material for: Hydrogen-Terminated Two-Dimensional Germanane/Silicane Alloys as Self-Powered Photodetectors and Sensors
Source: ACS Appl Mater Interfaces. 2023 May 16;15(21):25693–703. doi: 10.1021/acsami.3c01971 (PMC10236439; doi:10.1021/acsami.3c01971)
Supplement: Supplementary file 1 — am3c01971_si_001.pdf [file am3c01971_si_001.pdf]

Supporting Information

Hydrogen-terminated Two-dimensional Germananes/Silicanes Alloys as Self-powered Photodetectors and Sensors

Pradip Kumar Roy, *[a] *Tomáš Hartman,* [a] *Jiří Šturala* [a] *Jan Luxa,*[a] *Manuel Melle-Franco,*[b] *and Zdenek Sofer** [a]

[a] Department of Inorganic Chemistry, University of Chemistry and Technology Prague,

Technická 5, 166 28 Prague 6, Czech Republic

[b]CICECO − Aveiro Institute of Materials, Department of Chemistry, University of Aveiro,

3810-193 Aveiro, Portugal

E-mail: royp@vscht.cz, zdenek.sofer@vscht.cz

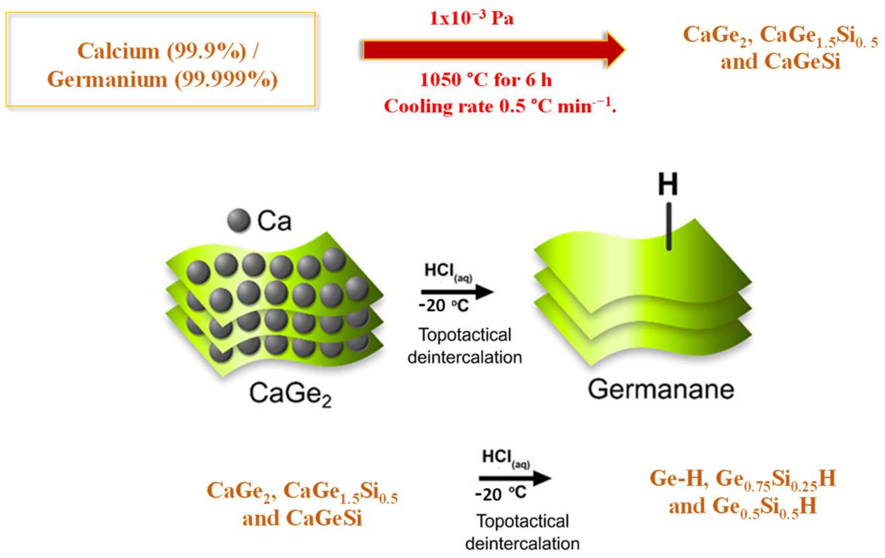

Figure S1. Schematic illustration of low temperature topochemical deintercalation.

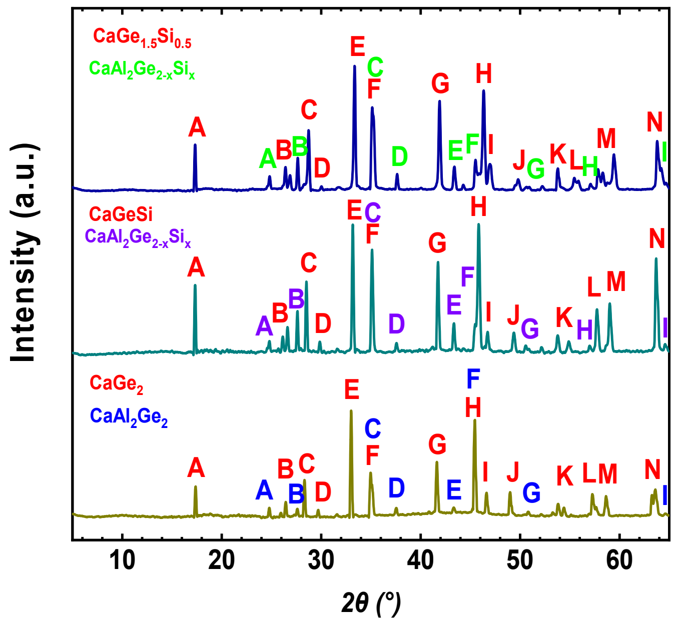

Figure S2. XRD of Zintl phases.

Table 1. XRD Peaks of $CaGe_2$ and $CaAl_2Ge_2$ with corresponding peak assignments

$CaAl_2Ge_2$				
LABEL	HKL	MEASURED	REF 04-011-0987	HKL
A	1 0 0	24.62	24.6	1 0 0
	0 0 2	24.78	24.804	0 0 2
B	0 1 1	27.61	27.603	0 1 1
C	0 1 2	35.21	35.215	0 1 2
D	0 0 3	37.55	37.586	0 0 3
E	1 1 0	43.3	43.311	1 1 0
F	1 0 3	45.43	45.44	1 0 3
G	0 0 4	50.82	50.882	0 0 4
	2 0 1	52.11	52.132	2 0 1
H	0 2 3	64.54	64.578	0 2 3

Table 2. XRD Peaks of $CaGe_{1.5}Si_{0.5}$ and $CaAl_2Ge_{2-x}Si_x$ with corresponding peak assignments

$CaGe_{1.5}Si_{0.5}$				$CaAl_2 Ge_{2-x} Si_x$		
LABEL	MEASURED	HKL		LABEL	MEASURED	HKL
A	17.33	0 0 6		A	24.63	1 0 0
B	26.42	1 0 1			24.82	0 0 2
	26.88	0 1 2		B	27.64	0 1 1
C	28.75	1 0 4		C	35.09	0 1 2
D	30.04	0 1 5		D	37.62	0 0 3
E	33.37	1 0 7		E	43.37	1 1 0
F	35.1	0 0 12		F	45.5	1 0 3
	35.22	0 1 8		G	50.62	1 1 2
G	41.91	0 1 11			52.18	2 0 1
H	46.35	1 1 0		H	57.06	2 0 2
I	46.9	1 0 13		I	64.66	0 2 3
J	49.85	1 1 6				
K	53.76	0 0 18				
L	55.44	2 0 2				
	55.89	0 2 4				
M	57.84	0 2 7				
	58.32	0 1 17				
	59.45	1 1 12				
N	63.81	**2 0 11**				
	64	**1 0 19**				

Table 3. XRD Peaks of CaGeSi and $CaAl_2Ge_{2-x}Si_x$ with corresponding peak assignments

CaGeSi				$CaAl_2$ Ge_{2-x} Si_x		
LABEL	MEASURED	HKL		LABEL	MEASURED	HKL
A	17.33	0 0 6		A	24.62	1 0 0
B	26.15	1 0 1			24.79	0 0 2
	26.64	0 1 2		B	27.62	0 1 1
C	28.5	1 0 4		C	35.29	0 1 2
D	29.86	0 1 5		D	37.56	0 0 3
E	33.19	1 0 7		E	43.32	1 1 0
F	35.03	0 0 12		F	45.44	1 0 3
	35.1	0 1 8		G	50.56	1 1 2
G	41.72	0 1 11			50.88	0 0 4
H	45.84	1 1 0			52.16	2 0 1
I	46.71	1 0 13		H	57.02	2 0 2
J	49.33	1 1 6			58.68	1 1 3
K	53.64	0 0 18		I	64.58	0 2 3
	53.92	2 0 2				
	54.94	0 2 4				
L	57.7	0 2 7				
	57.83	0 1 17				
M	59.01	1 1 12				
N	63.69	**2 0 11**				
	63.85	**1 0 19**				

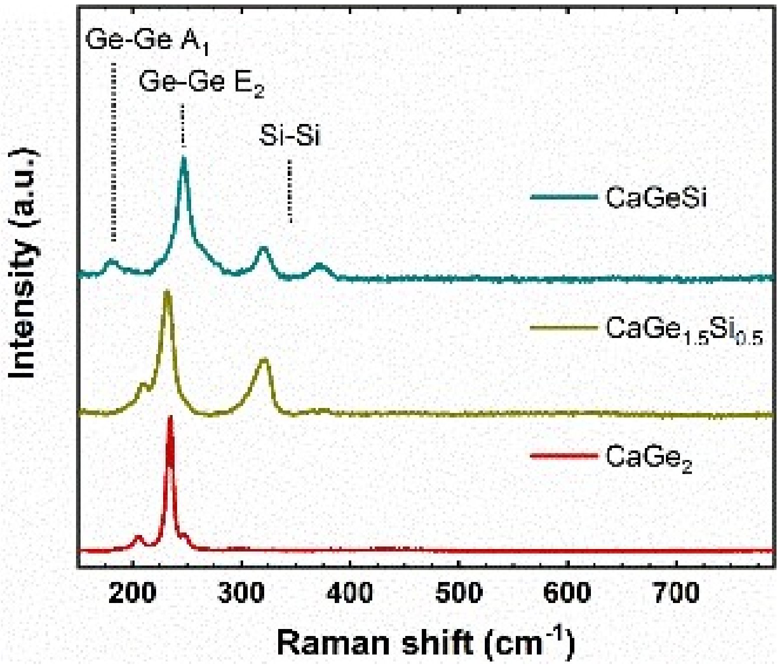

Figure S3. Raman Spectra of Zintl phases.

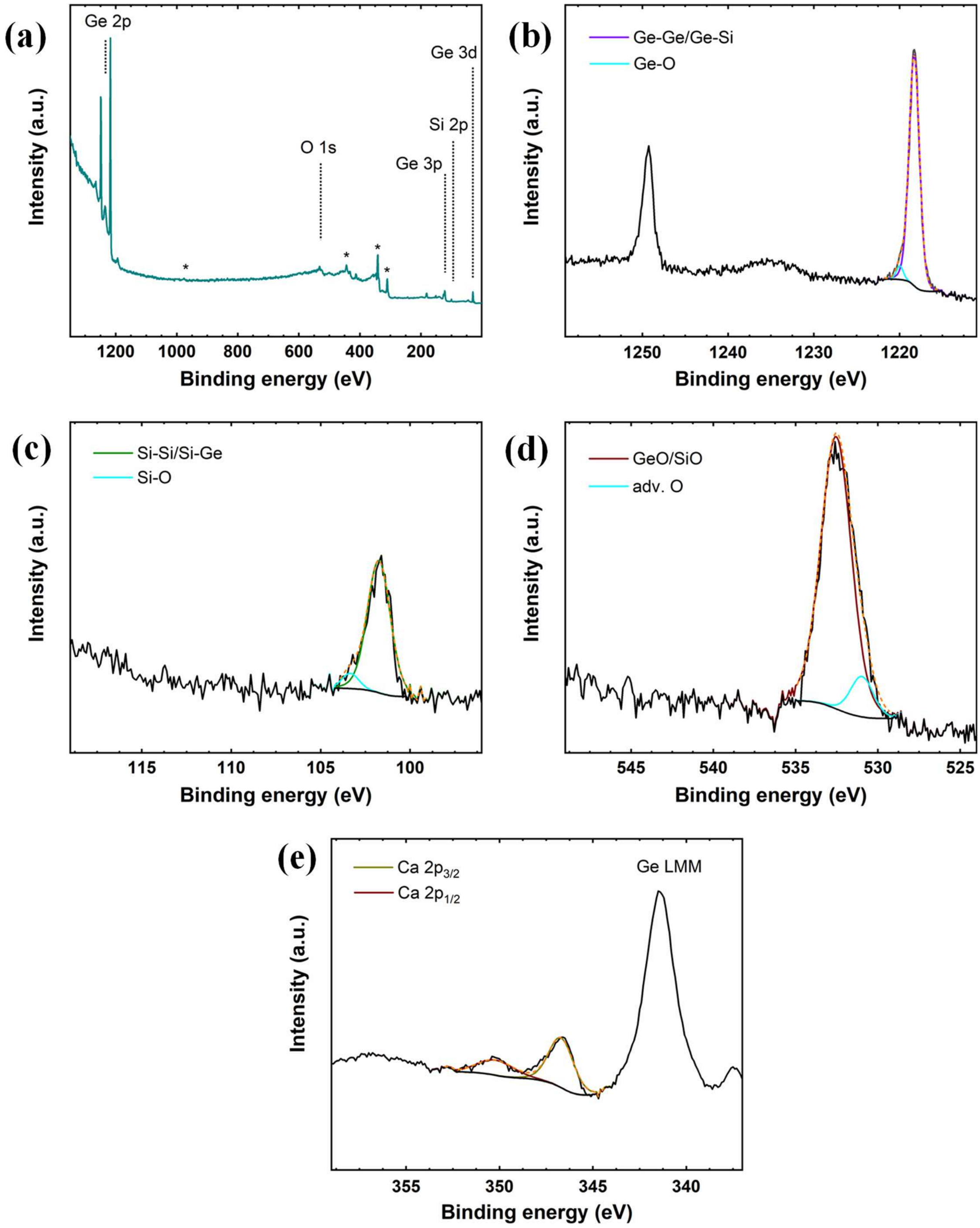

Figure S4. XPS spectra (a) survey and (b–e) High-resolution spectra of the corresponding elements of $CaGe_{1.5}Si_{0.5}$. Peaks marked with an asterisk are from the Si substrate.

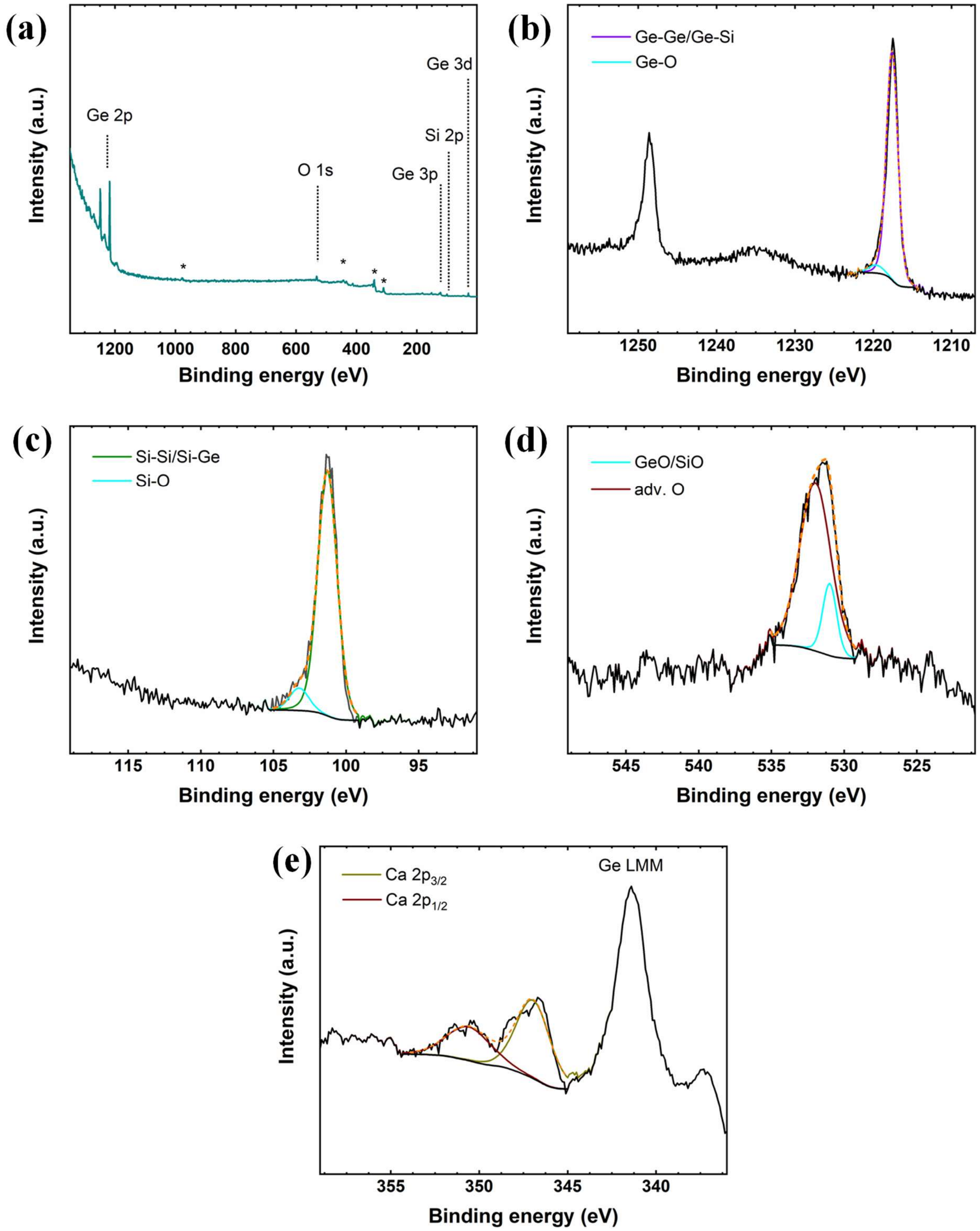

Figure S5. XPS spectra (a) survey and (b–e) High-resolution spectra of the corresponding elements of $CaGe_{1.0}Si_{1.0}$. Peaks marked with an asterisk are from the Si substrate.

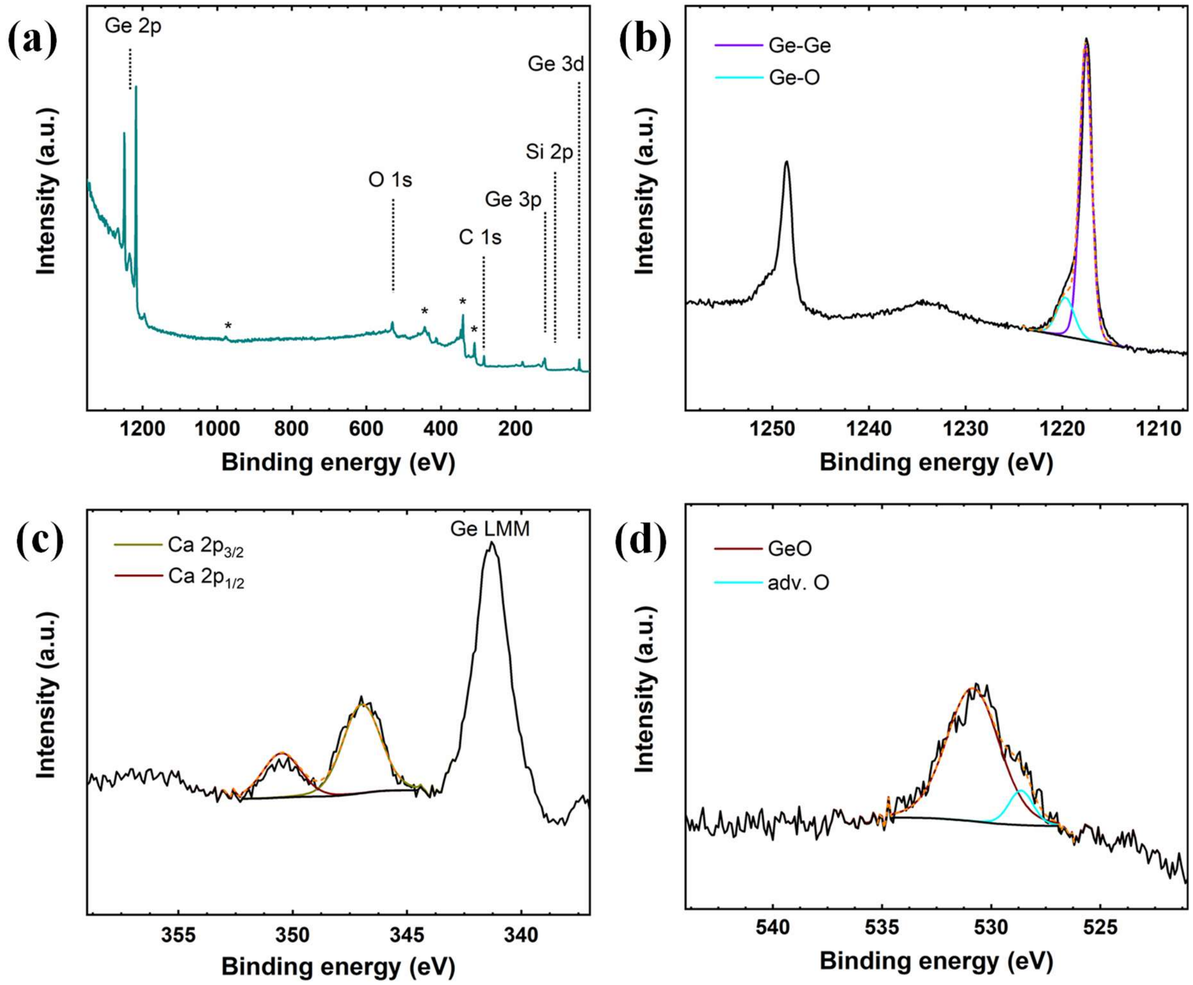

Figure S6. XPS spectra (a) survey and (b–d) High-resolution spectra of the corresponding elements of $CaGe_2$. Peaks marked with an asterisk are from the Si substrate.

GeH

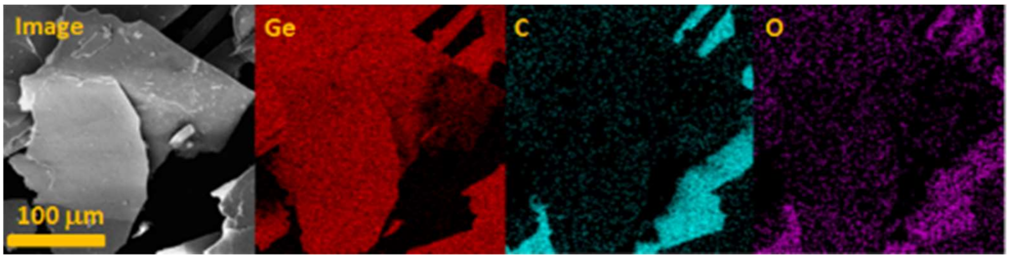

$Ge_{0.75}Si_{0.25}H$

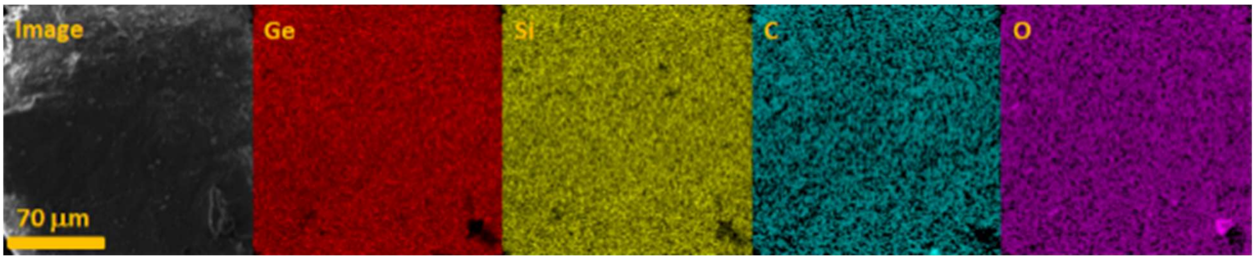

$Ge_{0.5}Si_{0.5}H$

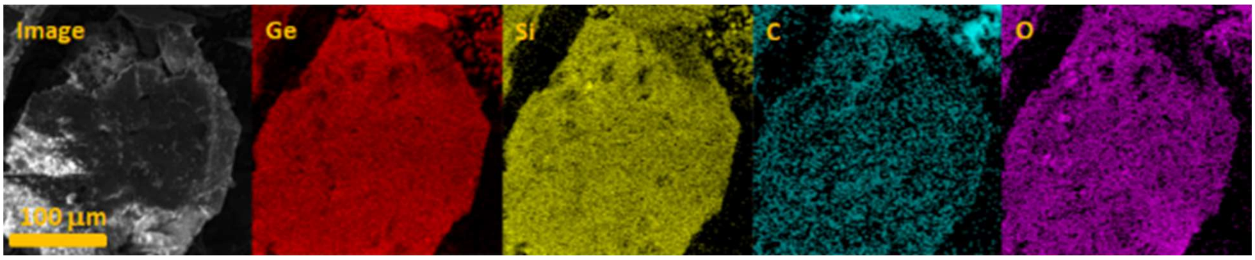

Figure S7. SEM and EDX of exfoliated Zintl phases show a uniform distribution of components across the materials.

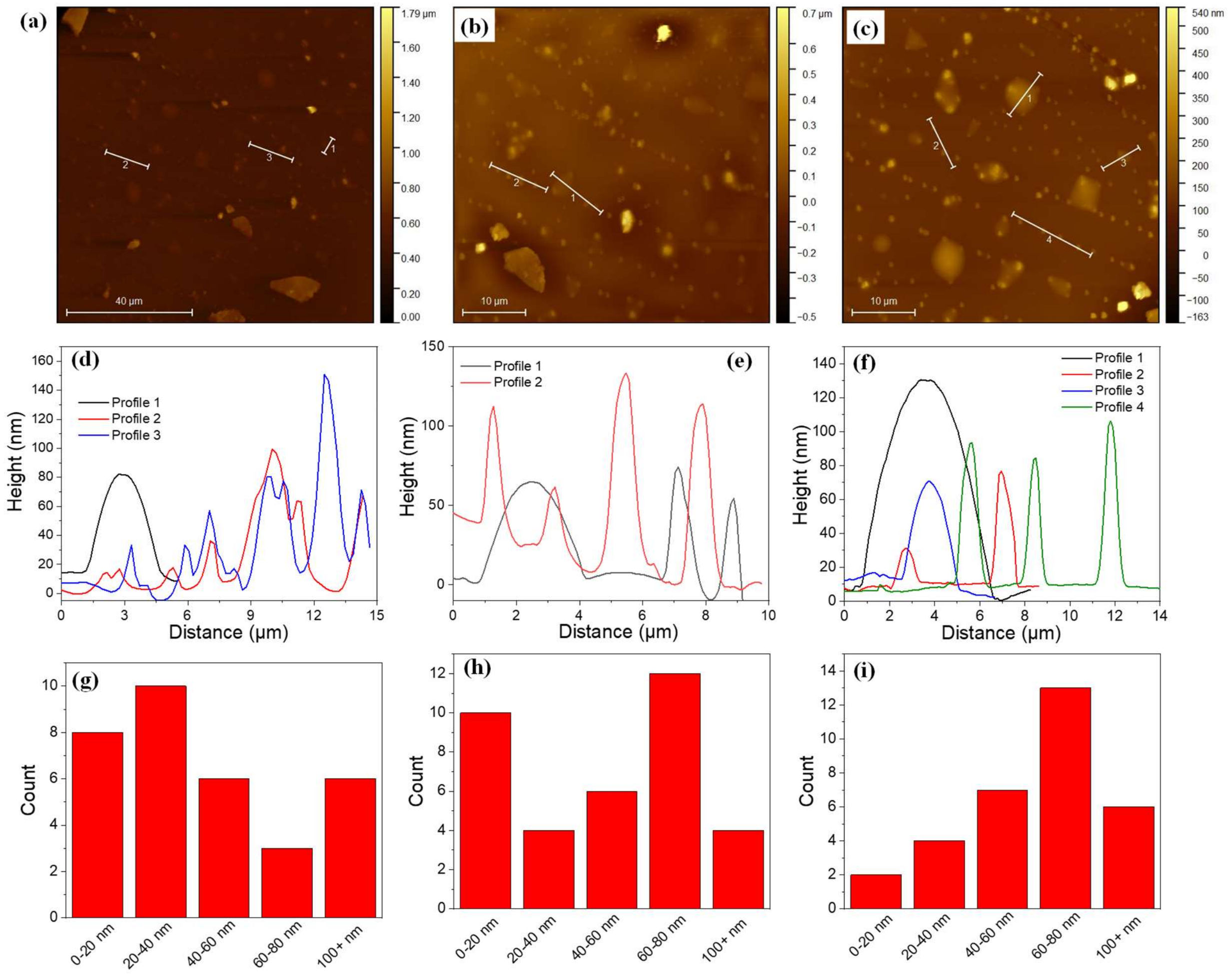

Figure S8. The flake thickness and corresponding thickness profile of the exfoliated Ge-H (a, d, g), $Ge_{0.75}Si_{0.25}H$ (b, e, h), and $Ge_{0.5}Si_{0.5}H$ (c, f, i).

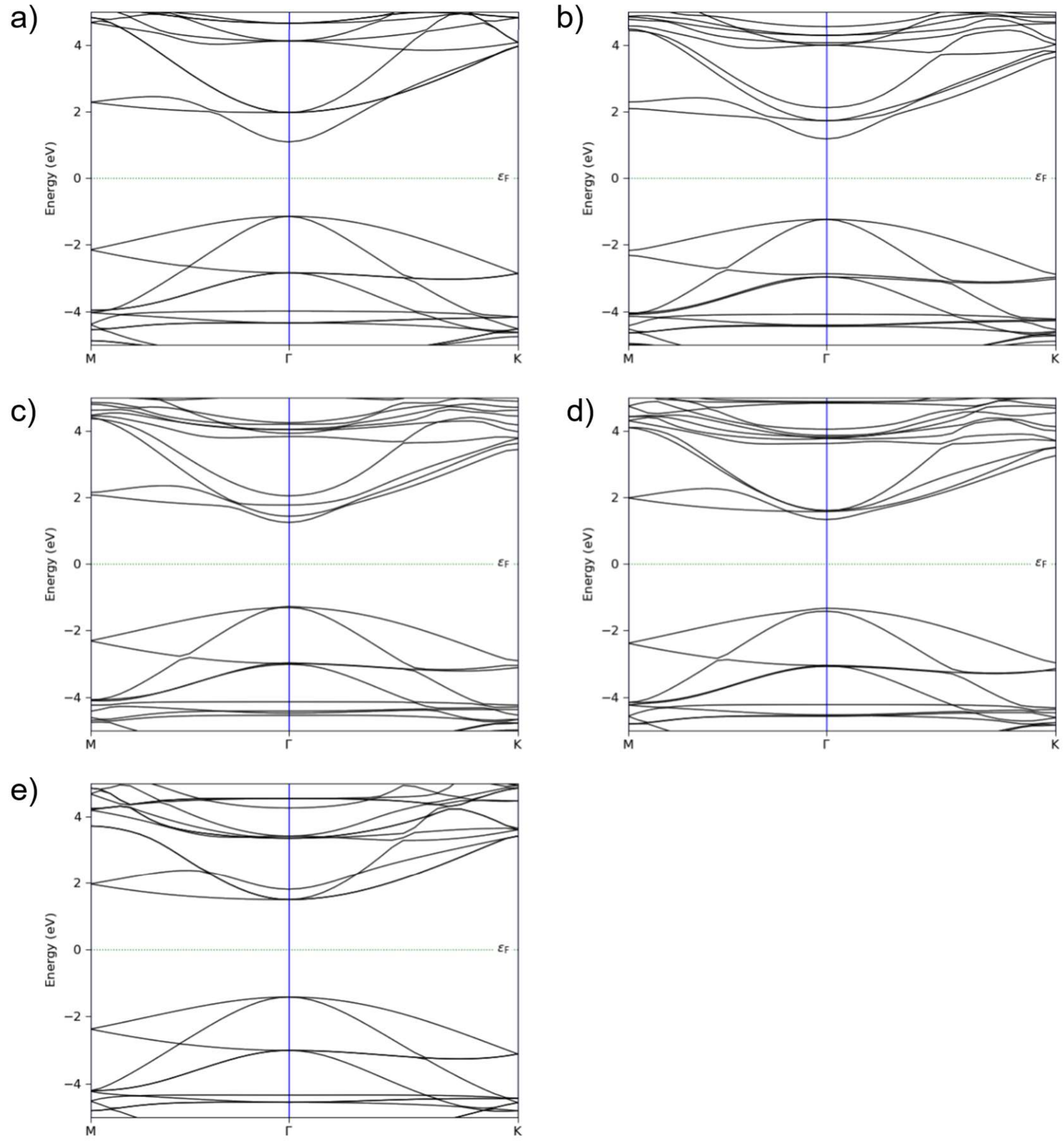

Figure S9. Computed band structures of (a) GeH, (b) $Ge_{0.875}Si_{0.125}H$, (c) $Ge_{0.75}Si_{0.25}H$, (d) $Ge_{0.5}Si_{0.5}H$ and (e) SiH.

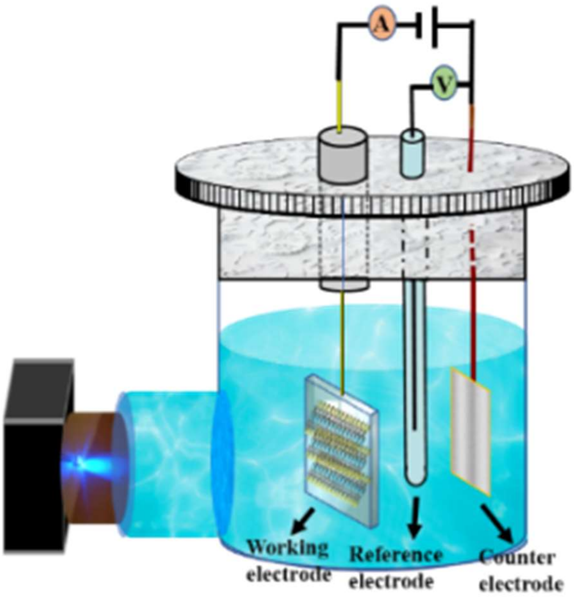

Figure S10. Experimental setup of a photoelectrochemical photodetector with a three-electrode system.

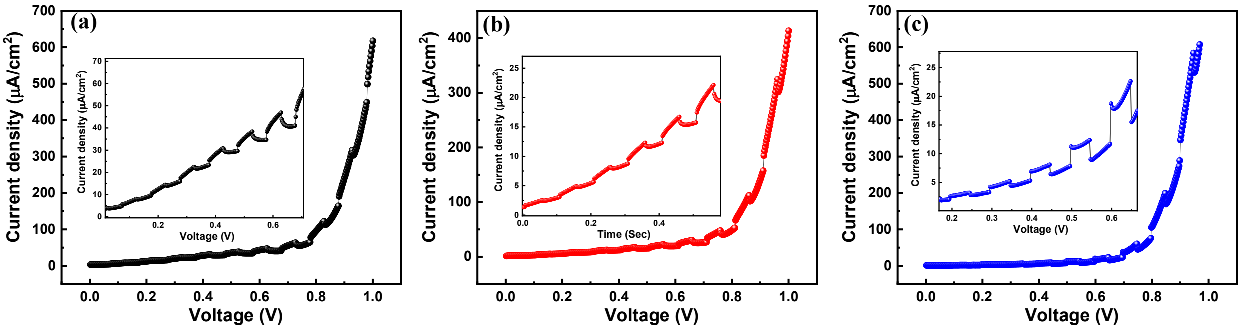

Figure S11. IV Characteristics of the (a) GeH, (b) $Ge_{0.75}Si_{0.25}H$ and (c) $Ge_{0.5}Si_{0.5}H$ under the illumination of 420 nm LED. The inset curve shows the magnified view of current density.

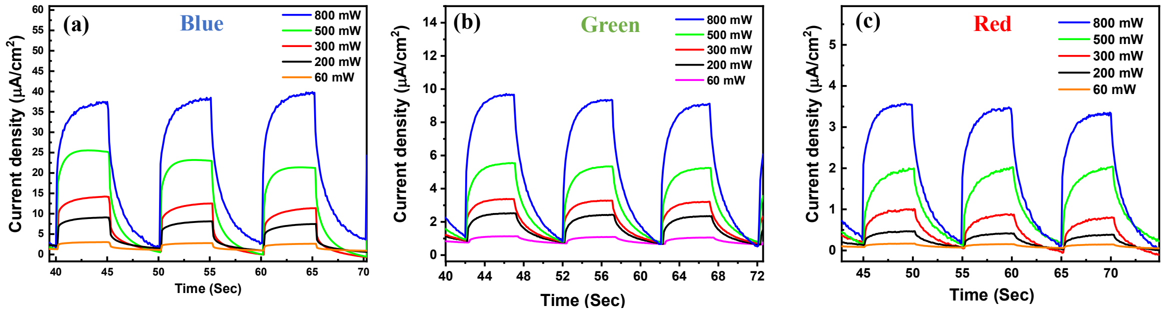

Figure S12. Photocurrent density at different powers (a) blue (b) green (c) red in 1 M KOH solutions for Ge-H.

Table S4. Comparison of our PEC-type photodetector with those of other solution-processed photodetectors reported in the literature.

Materials	Device configuration	Measurement conditions		Responsivity (mA W^{-1})	Wavelength (nm)	Reference
		Electrode	Applied Potential			
GaSe nanoflakes	PEC-type	0.5 M H_2SO4	-0.3 V *vs.* Ag/AgCl	160	455	*20*
				19.5	455	
InSe nanosheets	PEC-type	0.3 M KOH	0 V *vs.* SCE	10.14	365	*42*
GaSe nanoflakes	PEC-type	0.5 M H_2SO_4<br>1 M KOH	-0.05 V *vs.* RHE	320	455	*43*
In_2O_3 microrods	PEC-type	0.5 M H_2SO_4<br>1 M KOH	0.6 V *vs.* Ag/AgCl	21.19	365	44
InSe nanosheets	PEC-type	0.2 M KOH	1 V *vs.* SCE	3.3×10^{-3}	Simulated sunlight	*45*
				4.9×10^{-3}	Simulated sunlight	
Black phosphorous nanosheets	PEC-type	0.1 M KOH	0 V *vs.* SCE	1.9×10^{-3}	Simulated sunlight	*46*
				2.2×10^{-3}	Simulated sunlight	
Perovskite ($CH_3NH_3PbI_3$)	Metal-semiconductor-metal	0.1 M KOH	5 V	4.4	633	*47*
SnS	PEC-type	0.1 Na_2SO_4	0.6 V	0.018	365	*48*
GeSe nanosheets	PEC-type	0.1 M KOH	0.3 V	0.044	Simulated sunlight	*49*
				0.076	Simulated sunlight	
PBDTT-ffQx/PCBM bulk heterojunction	Metal-semiconductor-metal	-	10 V	1.15×10^3	365	*50*
SnS/RGO hybrid nanosheets	FET	-	V_{DS} =5V, V_g = 0 V	180	visible light	*51*
**GeH**	**PEC-type**	**1 M KOH**	**0.5 V *vs.* SCE**	**0.168**	**420 nm**	**This Work**
		**0.5 H_2SO_4**	**-0.5 V *vs.* SCE**	**0.056**	**420 nm**	

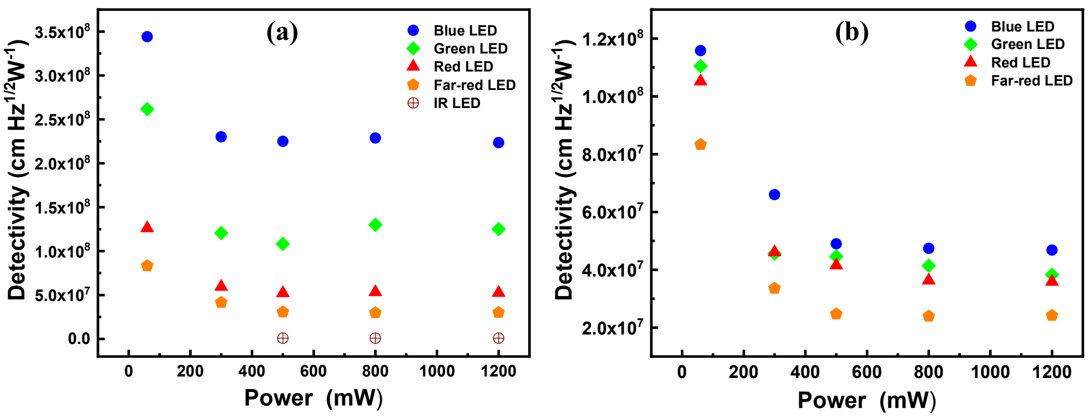

Figure S13. The Detectivity of PEC-type photodetectors in (a)1 M KOH solution (b) 0.5 M H_2SO_4 solution as a function of power with different illumination wavelengths.

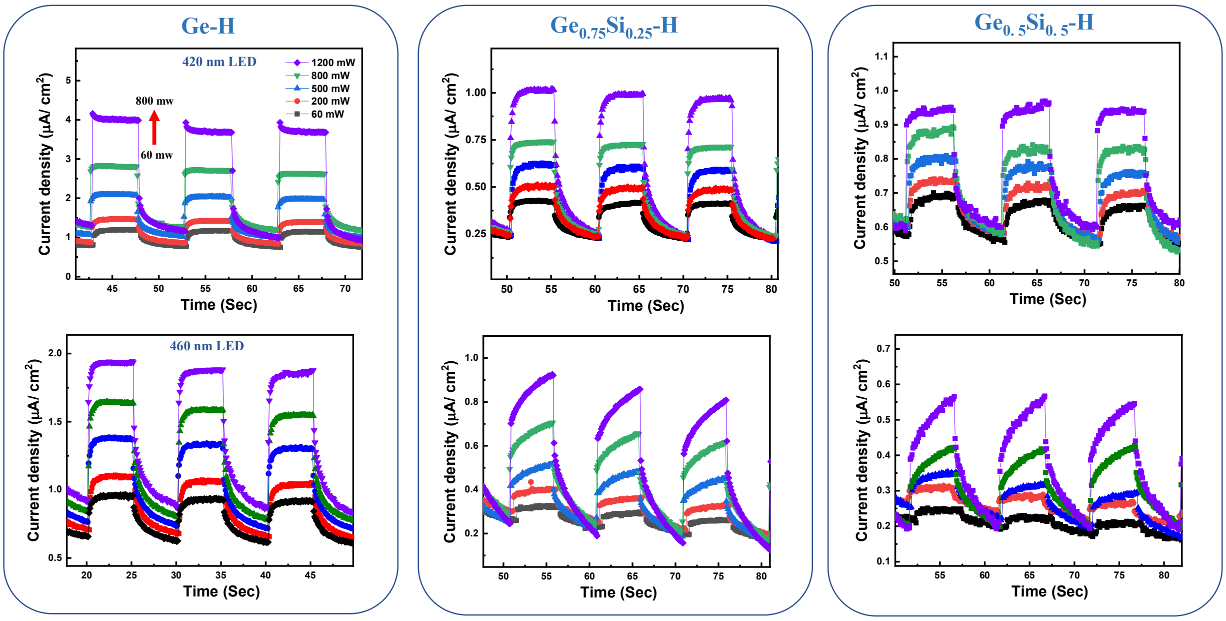

Figure S14. Photocurrent density with different wavelengths and powers in 0.5 M Na_2SO_4 solutions for Ge-H, $Ge_{0.75}Si_{0.25}H$ and $Ge_{0.5}Si_{0.5}H$.

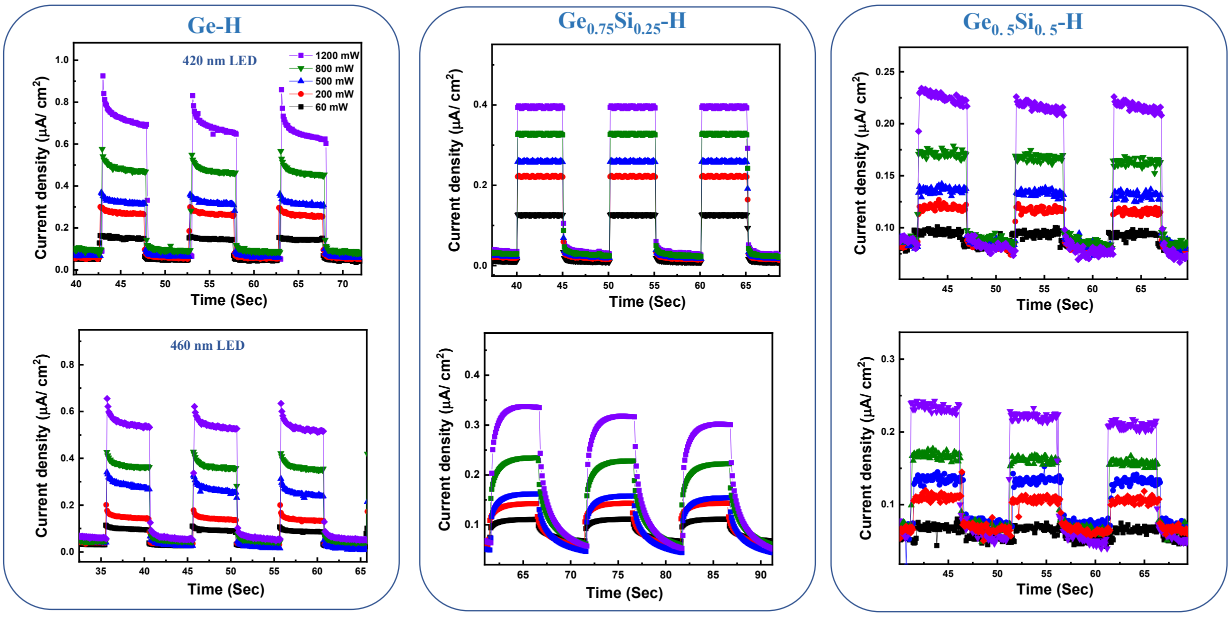

Figure S15. Photocurrent density with different power and wavelength in ionic solutions for Ge-H, $Ge_{0.75}Si_{0.25}H$ and $Ge_{0.5}Si_{0.5}H$.

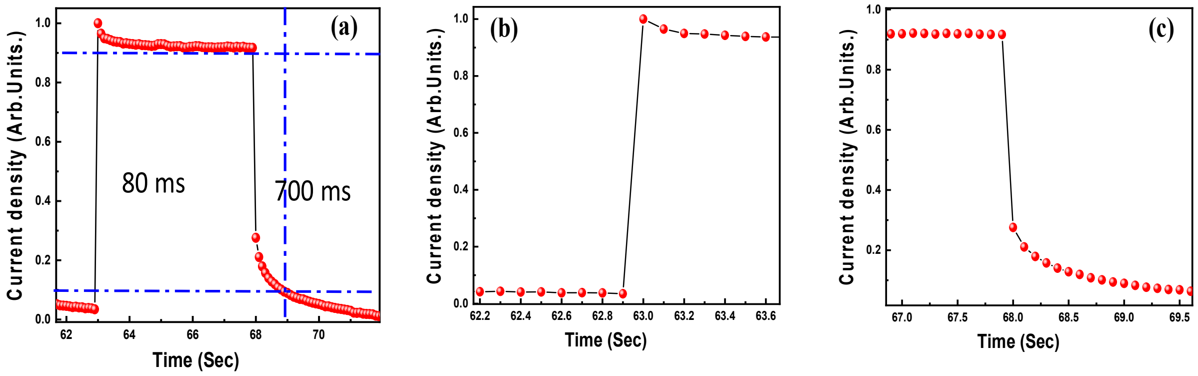

Figure S16. (a–c) Response time of the photodetector device in Na_2SO_4 solutions for Ge-H.

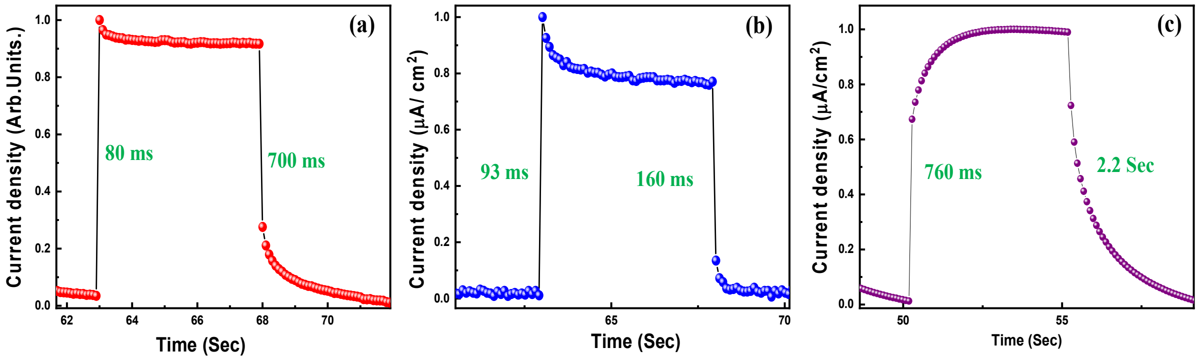

Figure S17. Comparison of the response time of the photodetector device in (a) Na_2SO_4 (b) ionic (c) KOH solutions for Ge-H.

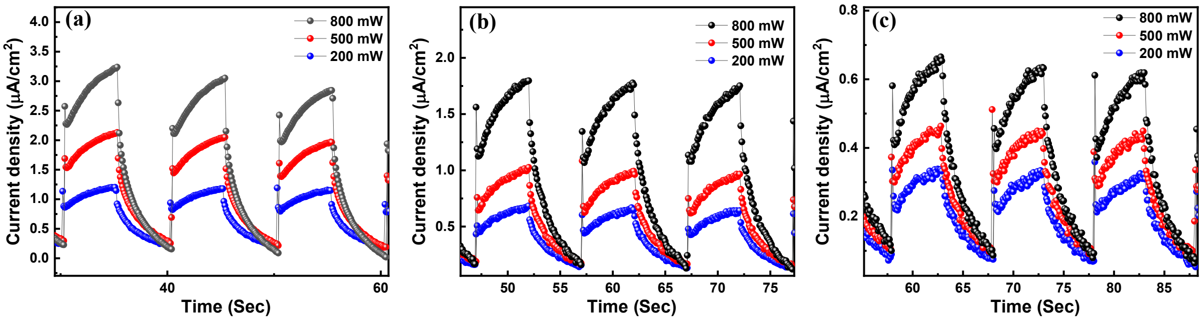

Figure S18. Photocurrent density of Ge-H at an applied voltage of 0 V vs SCE with (a) 420 nm (b) 460 nm and (c)532 nm LED illumination.

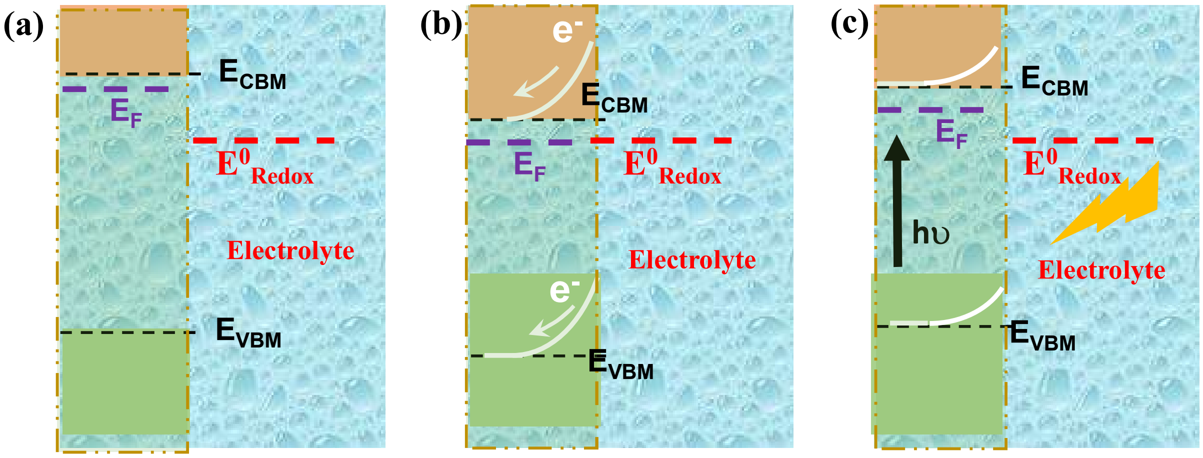

Figure S19. Band bending and self-powered mechanism (a) before immersed in the solution (b) after immersion in solution (c) under the illumination of 420 nm LED.

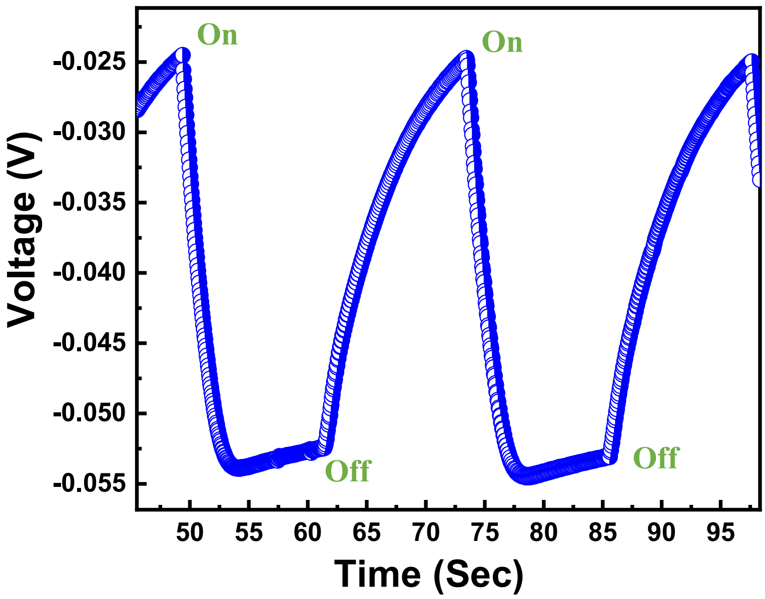

Figure S20. Open circuit potential under the illumination of 420 nm LED (800 mW).

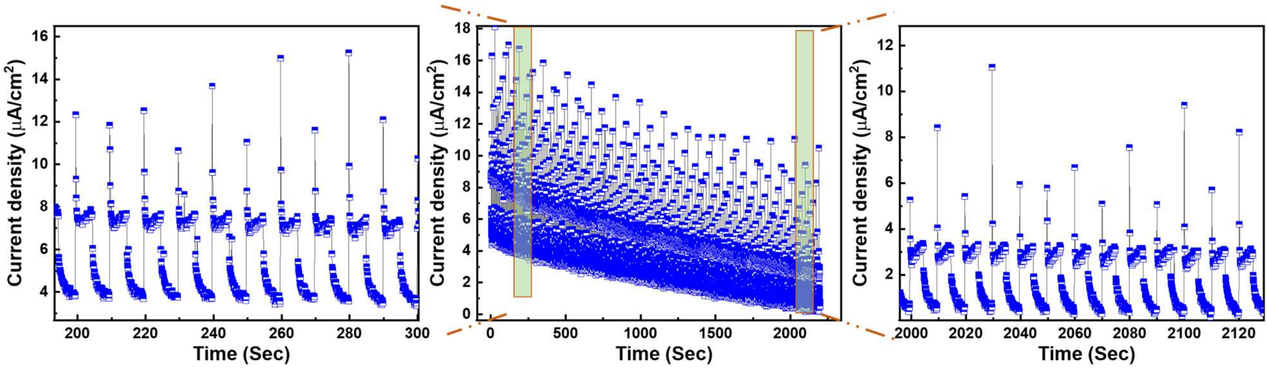

Figure S21. Photo response and "on-off" switching behaviour under the illumination of a 420 nm LED light source (500 mW power).

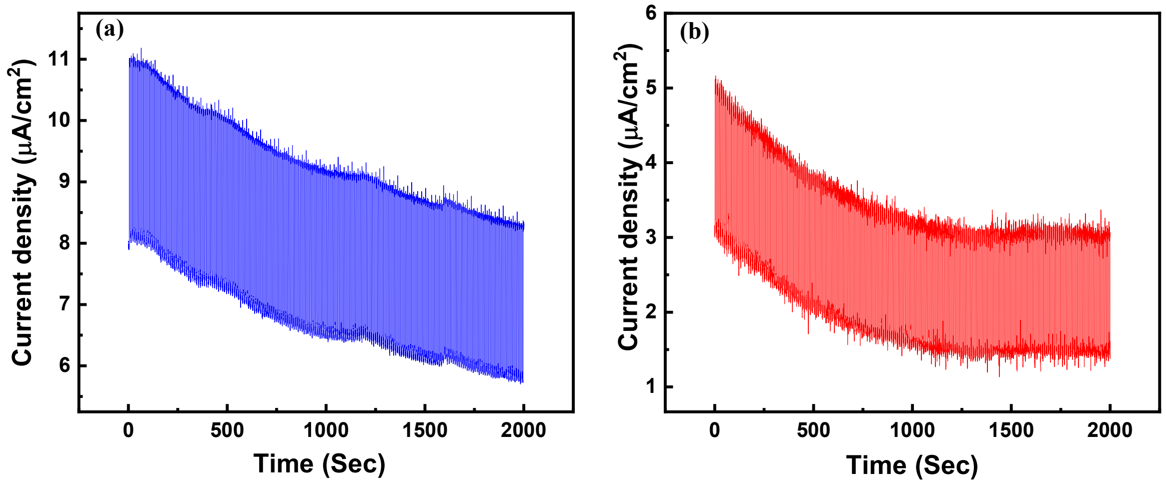

Figure S22. Photo response and "on-off" switching behaviour under the illumination of a 420 nm LED light source (500 mW power) (a) $Ge_{0.75}Si_{0.25}H$ and (b) $Ge_{0.5}Si_{0.5}H$.

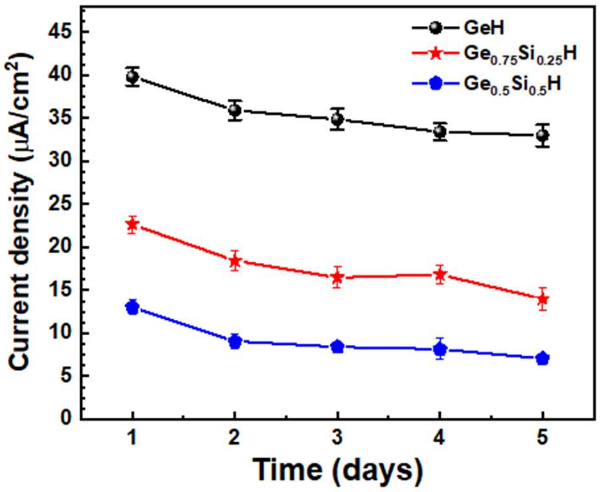

Figure S23. long-term stability of the photodetector: Photocurrent density of (a) Ge-H (b) $Ge_{0.75}Si_{0.25}H$ and (c) $Ge_{0.5}Si_{0.5}H$ at an applied voltage of 0.5 V vs SCE with time.

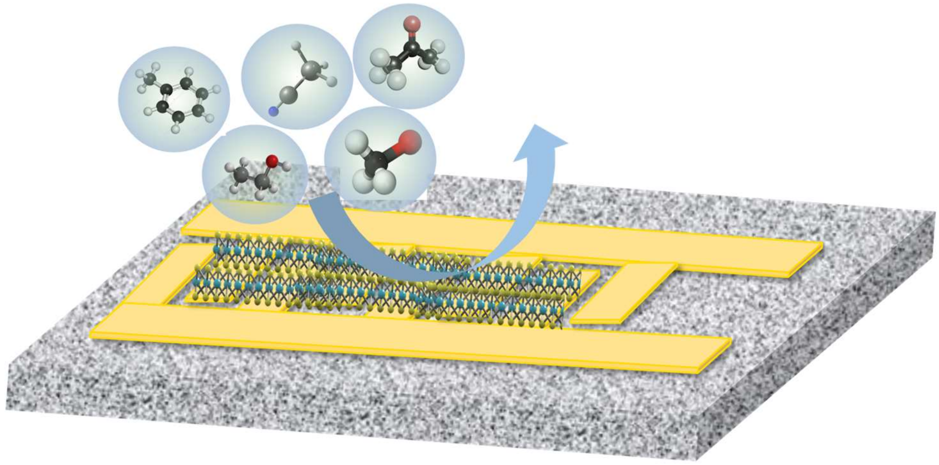

Figure S24. Schematic illustration of vapor sensor device.

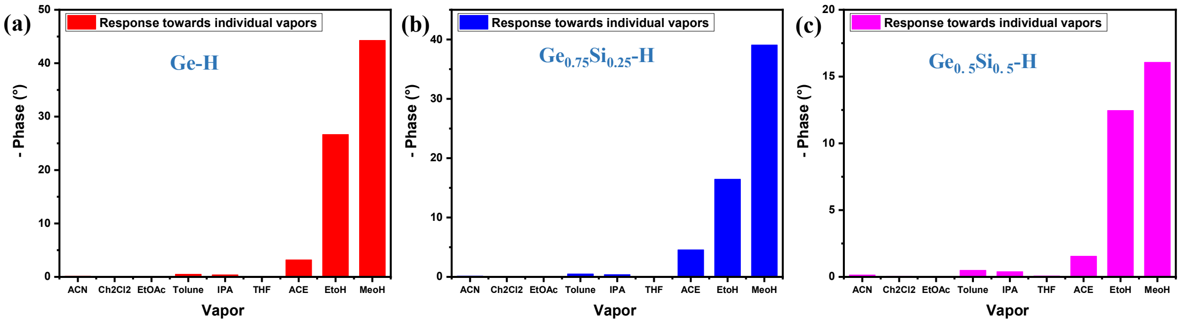

Figure S25. Response of individual solvent vapours towards the sensor devices (a) Ge-H, (b) $Ge_{0.75}Si_{0.25}H$ and (c) $Ge_{0.5}Si_{0.5}H$.

.

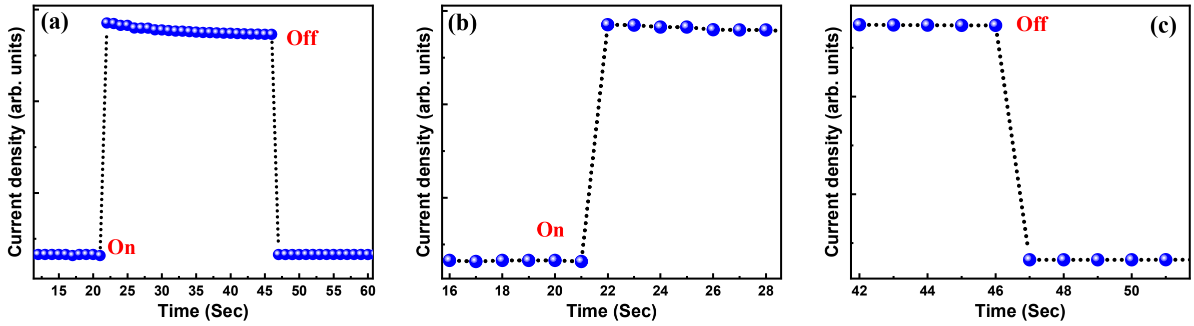

Figure S26. (a–c) Response time of the vapor sensor.

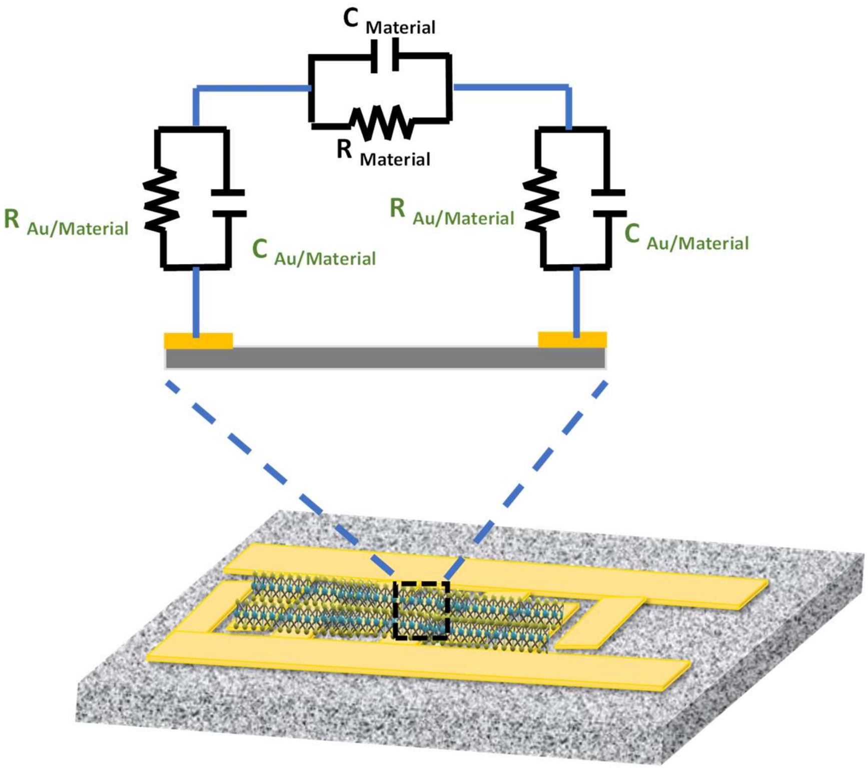

Figure S27. RC equivalent circuit of the sensor device.

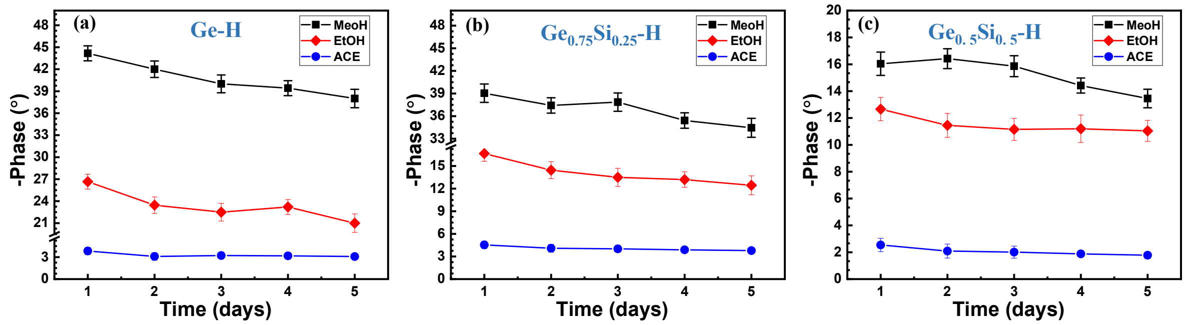

Figure S28. Stability of the sensor devices (a) Ge-H, (b) $Ge_{0.75}Si_{0.25}H$ and (c) $Ge_{0.5}Si_{0.5}H$.
